# Traits, landmarks and outlines: Three congruent sides of a tale on coral reef fish morphology

**DOI:** 10.1002/ece3.8787

**Published:** 2022-04-21

**Authors:** Marita Quitzau, Romain Frelat, Vincent Bonhomme, Christian Möllmann, Leopold Nagelkerke, Sonia Bejarano

**Affiliations:** ^1^ Aquaculture and Fisheries Group Wageningen Institute of Animal Sciences Wageningen University and Research Wageningen The Netherlands; ^2^ UMR 5554 Institut des Sciences de l’Evolution, équipe Dynamique de la biodiversité Anthropo‐écologie Université de Montpellier CNRS IRD Montpellier Cedex 05 France; ^3^ Centre for Earth System Research and Sustainability (CEN) Institute of Marine Ecosystem and Fishery Science University of Hamburg Hamburg Germany; ^4^ Reef Systems Research Group Ecology Department Leibniz Centre for Tropical Marine Research Bremen Germany

**Keywords:** coral reefs, fish morphology, geometric morphometrics, landmark analysis, outline analysis, trait‐based approaches

## Abstract

Quantifying the morphology of organisms remains fundamental in ecology given the form‐function relationship. Morphology is quantifiable in traits, landmarks, and outlines, and the choice of approach may influence ecological conclusions to an unknown extent. Here, we apply these three approaches to 111 individual coral reef fish of 40 species common in Micronesia. We investigate the major dimensions of morphological variability among individuals, families, and predefined feeding functional groups. We find that although the approaches are complementary, they coincide in capturing elongation as the main dimension of variability. Furthermore, the choice of approach led to different interpretations regarding the degree of morphological differentiation among taxonomic and feeding functional groups. We also use each morphology dataset to compute community‐scale morphological diversity on Palauan reefs and investigate how the choice of dataset affects the detection of differences among sites and wave exposure levels. The exact ranking of sites from highest to lowest morphological diversity was sensitive to the approach used, but not the broad spatial pattern of morphological diversity. Conclusions regarding the effect of wave exposure on morphological diversity were robust to the approach used. Biodiversity hotspots (e.g., areas of exceptionally high diversity and/or endemism) are considered important conservation targets but their location may depend on the biodiversity metric used. In the same vein, our results caution against labelling particular sites as morphological diversity hotspots when metrics consider only a single aspect of morphology.

## Graphical abstract

We provide a direct comparison among traditional morphometrics, landmark analysis, and outline analysis applied to a common pool of coral reef fish species. Although all approaches identified the extent of body elongation as the main source of variation among individual fish, the choice of approach led to different interpretations regarding the degree of disparity among taxonomic and feeding functional groups. All approaches, however, arrived at congruent ecological conclusions regarding the spatial variability of morphological diversity of coral reef fish.

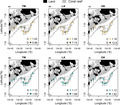

## 1 INTRODUCTION

The study of organisms’ morphology remains a fundamental task in ecology given the close relationships among form, function, evolution, and the environment (Lauder, 1990; Thompson, 1917). Investigating whether distinct morphological features are consistently associated with different taxonomic groups and/or functional roles is key to clarifying the extent to which phylogenetic history or adaptive evolution shapes current ecological diversity (Pigot et al., 2020). The integration of species morphology and occurrence data is fundamental in ecology because it holds the potential of revealing spatial patterns of morphological diversity and how these relate to environmental variables. This is key to understanding the complex processes contributing to evolution and diversification, disentangling natural and anthropogenic drivers of global biodiversity, and assessing the vulnerability of biogeographic realms to species loss (Norris et al., 2021; Su et al., 2019; Toussaint et al., 2016). Several morphological attributes are strongly linked to species feeding modes and functions (Villeger et al., 2017; Wainwright & Bellwood, 2002). Thus, the morphological diversity of ecological communities covaries with their functional diversity (Schneider et al., 2017; Sol et al., 2020). Functional diversity is a recognized proxy for ecosystem functioning and stability, both of which are crucial for the long‐term provisioning of ecosystem services (Díaz & Cabido, 2001; Villéger et al., 2014).

Three methodological approaches are commonly used to characterize the morphology (i.e., size and shape) of organisms. Traditional morphometrics (TM) describes morphology using ratios between lengths, angles, and areas measured on body parts (Bellwood et al., 2014; Gatz, 1979). These ratios (i.e., morphological traits), often correlate with functions such as locomotion or diet (Sibbing & Nagelkerke, 2001; Webb, 1984). Geometric morphometrics approaches, namely landmark analysis (LA) and outline analysis (OA), directly capture geometry which can be, by construction, separated into size and shape. LA is based on the positions of landmarks, defined as homologous points common to all individuals within a population (Farré et al., 2016; Mitteroecker & Gunz, 2009). Unlike TM, LA preserves the relationships among landmarks and their geometry. Both LA and TM, however, depend on available landmarks and traits, and on the likely subjective choice of whether to include them or not in a particular analysis (Farré et al., 2016). In contrast, OA describes the entire geometry of the outline of organisms through mathematical functions, hence circumventing any bias linked to a priori selection of traits or landmarks (Bonhomme et al., 2014; Claude, 2008). However, OA excludes features that fall without the outline (e.g., pectoral fins or eyes), thus missing particular shape information that can be captured by TM and LA. Therefore, by their very nature, each approach has the potential to capture different aspects of morphological diversity.

Which morphometric approach is used in ecological research depends on the aims and taxa under consideration, but might also be influenced by the popularity of methods within different scientific fields. OA, for instance, is often used to study the shape of objects with limited homologous points, such as otoliths (Mérigot et al., 2007) or pollen grains (Bonhomme et al., 2013), but it is inappropriate when the shape of the objects under investigation are poorly preserved (e.g., fish fins in museum specimens). LA is primarily applied to characterize the morphology of the skeleton or skull of vertebrates, with numerous homologous points and complex outlines (Ibañez et al., 2007; Maestri et al., 2018), while TM is currently often used to quantify morphological traits with a functional interpretation (e.g., Sibbing & Nagelkerke, 2001). The rationale behind the choice of method is, however, not always obvious. To characterize fish morphology, some studies used TM (Bejarano et al., 2017; Bellwood et al., 2014; Villéger et al., 2010; Winemiller, 1991), others LA (Claverie & Wainwright, 2014a; Costa & Cataudella, 2007; Elmer et al., 2010; Klingenberg et al., 2003), and more recent ones OA (Caillon et al., 2018; Ventura et al., 2017). Given that TM, LA, and OA capture different aspects of morphology, applying one or the other on the same pool of organisms may highlight different dimensions of morphological disparity among individuals, taxonomic groups, or predefined functional groups. Moreover, the extent to which combining the morphology datasets derived from TM, LA, and OA with the same *species occurrence* × site matrix (e.g., Su et al., 2019) may lead to different community‐level estimates of morphological diversity, is yet to be quantified. Biologists and ecologists should thus understand the scope, and be aware of the potential effects of using any given morphometric approach in order to adequately address questions on shape diversity and ecological functioning. However, a study applying the three approaches on the same multispecies assemblages is currently lacking, yet could reveal which aspects of shape are picked up by each of the methods and how this relates to taxonomy or trophic ecology (Kerschbaumer & Sturmbauer, 2011).

Tropical coral reefs promote the evolution of morphological diversity and trophic novelty (Price et al., 2011). Coral reef fish constitute one of nature's most striking cases of morphological diversity (Claverie & Wainwright, 2014b) while sustaining ecosystem functions and services that are pivotal for the oceans and humanity (Villéger et al., 2017). Nominally herbivorous reef fish, for example, display a remarkable intra‐guild variety of shapes and feeding behaviors that mediate the competitive balance between corals and algae (Siqueira et al., 2019), thus contributing to the resilience of coral reefs to perturbations (Bellwood et al., 2004; Nash et al., 2016). A range of coarse to fine classification systems have been designed to collapse herbivorous fish species into feeding functional groups with complementary roles in preconditioning reefs for coral recovery (Bejarano et al., 2019; Bellwood et al., 2004; Green & Bellwood, 2009; Siqueira et al., 2019). Understanding how herbivorous fish individuals, species, and feeding functional groups differ in morphology is important from a biomechanics and evolutionary perspectives (Larouche et al., 2020). With TM, coarse feeding functional groups identified in pre‐20th century reefs (grazer‐detritivores vs. scrapers), differ in morphological features correlated with swimming performance (Bejarano et al., 2017). The degree to which more recent functional categorizations considering *how* and *what* species feed *on* (Bejarano et al., 2019; Bellwood et al., 2019; Siqueira et al., 2019) differ in morphology is less well understood. Moreover, whether different dimensions of morphological similarity or disparity among groups can be found when morphology is characterized using landmarks or outlines, remains to be examined. Morphology dictates how organisms interact with their environment at least partially determining the environmental contexts in which they are successful (Zawada et al., 2019). Thus, morphologically diverse species assemblages are more likely to exhibit broad ranges of functions and tolerance to disturbance (Madin & Connolly, 2006). Identifying areas of exceptionally high community‐scale morphological herbivore biodiversity in local contexts (i.e., morphological diversity hotspots) may therefore be relevant to support effective management strategies aimed at securing herbivory in times of environmental change and species loss (Beger et al., 2003; Craven et al., 2018; Robinson et al., 2014; Stuart‐Smith et al., 2013). Morphological diversity (correlated to swimming performance) of herbivorous reef fish (computed based on TM) in a forereef in Palau decreases with increasing wave exposure (Bejarano et al., 2017). Whether the different facets of morphological diversity of these assemblages captured by LA and OA respond equally to wave exposure remains untested.

Here, we assessed the morphology of nominally herbivorous reef fish using traits (TM), landmarks (LA), and outlines (OA) and asked how congruent the ecomorphological conclusions obtained with these approaches are. To this aim, we used 111 photographs of adult individual fish of 40 species commonly found on Micronesian coral reefs which are located in the vicinity of the Coral Triangle, a hotspot of global biodiversity. We quantified the degree of morphological disparity among individuals, genera, families, and a priori‐defined feeding functional groups, ordering individuals in three multidimensional spaces (hereafter referred to as morphospaces) based on their dissimilarity in traits, landmarks, and outlines. Specifically, we (a) identified the major dimensions of variation among individuals in all three aspects of morphology, (b) determined which morphological characteristics correlate strongly with taxonomic and a priori‐defined feeding functional groups, and (c) tested whether used in combination with in situ estimates of species occurrence, the three morphology datasets uncover different spatial patterns of morphological diversity over a 20‐km forereef in Palau.

## 2 MATERIALS AND METHODS

### 2.1 Overview of the procedure

This study focused on nominally herbivorous fish commonly observed on Micronesian forereefs (Mumby et al., 2013) and took place in three stages. First, we compiled morphological information on photographs for 111 individuals from 40 species representing 11 genera within three families (i.e., Acanthuridae, Siganidae, Labridae: Scarinae, Table 1). Second, we assigned each of the 40 species to predefined feeding functional groups that contribute differently to (i) bioerosion (*n* = 3), (ii) algal turf removal (*n* = 4), and (iii) macroalgal removal (*n* = 3; Table 1; Bejarano et al., 2019). Feeding functional groups were predefined independently of morphology, based on an exhaustive literature review of ~3000 published scientific papers documenting *how* fishes feed and *what* components of the reef benthos they interact with when feeding (Table 1; Bejarano et al., 2019). We concentrate on fish interactions with the calcareous reef matrix, algal turfs, and macroalgae because these underpin three ecosystem functions critical in maintaining the integrity of coral reefs and influencing post‐disturbance recovery (i.e., bioerosion, algal turf removal, and macroalgal removal; Table S2). Third, applying a trait‐based approach, we combined the morphology data with in situ occurrence data collected for these 40 species during a field study conducted by one of the authors (SB) in 2012 in the Palau Archipelago (i.e., Bejarano et al., 2017). The Palau Archipelago is located ~360 km east of the Philippines, and thus adjacent to the Coral Triangle Region, which contains 37% of the world's reef fishes and comprises a global hotspot of marine biodiversity (Allen, 2008; Veron et al., 2009). Species occurrence (presence–absence) data were derived from video recordings conducted at 12 shallow (6.8 ± 0.3 m) sites distributed over a 20‐km‐long section of the Eastern barrier reef situated approximately 10 km off the post‐populated island of Koror (Bejarano et al., 2017). According to prevailing wind direction the 12 sites were stratified across three levels of wave exposure namely *low* ranging from 0.9 to 23.6 J/m^3^ (*n* = 4), *moderate* from 46.7 to 72 J/m^3^ (*n* = 5), and *high* at, ~220 J/m^3^ (*n* = 3; Table S3). Wave exposure was quantified using a wave‐theory GIS approach (Chollett & Mumby, 2012) that integrates spatial information on the coastline and reef crests (Battista et al., 2007), as well as data on wind direction and speed at the time of the surveys (Feb–Mar 2012; Bejarano et al., 2017).

**TABLE 1:**
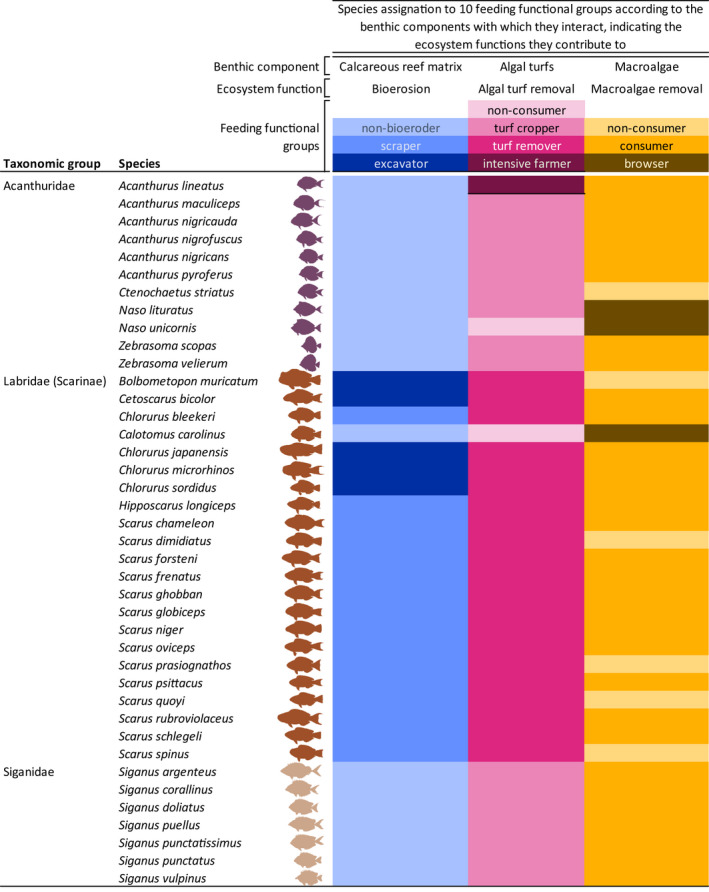
Species considered in this study indicating their assignation to 10 feeding functional groups

Note

Feeding functional groups were defined here according to *how* species interact with four different key components of the benthos, namely the calcareous reef matrix, algal turfs, detritus, and macroalgae (i.e., *what* sensu Bellwood et al., 2019). Assignations were determined through a systematic literature review encompassing ~3000 published papers or reviews (for details see Bejarano et al., 2019).

### 2.2 Morphometrics approaches

Morphology was quantified using three morphometrics approaches on the 111 photographs compiled by Bejarano et al. (2017). Each photograph contained a pinned specimen of known size, and to control for possible effects of allometry we focused on adult fishes. Images were downloaded from FishBase (Froese & Pauly, 2019) and the repository of photographs taken by Professor John Randall at the Bishop museum (http://pbs.bishopmuseum.org/images/JER/images.asp).

For TM, we measured 13 numerical morphological traits directly related to diet and locomotion (Table S1; Figure S1). We chose these traits for our comparative purpose because they have been extensively used in functional ecology studies to investigate, for instance, the impact of human disturbances on ecosystem functioning (Villéger et al., 2010), functional innovations along evolutionary history (Bellwood et al., 2014), or environmental filtering across localized environmental gradients (Bejarano et al., 2017). The traits were derived from 17 lengths and three areas measured per individual (Figure S1).

For LA, we located 12 landmarks commonly used to study fish morphology (Claverie & Wainwright, 2014a; Costa & Cataudella, 2007; Elmer et al., 2010; Klingenberg et al., 2003) on each image in our collection (Figure S2). Landmarks corresponded to homologous points found in all images which identify key evolutionary or functional features (Figueirido et al., 2010; Farré et al., 2013). We digitized the (*x*, *y*) coordinates per landmark and image with ImageJ 1farf.52 (http://imagej.nih.gov/ij/), and standardized the resulting landmark configurations using a full generalized Procrustes alignment, which superimposes landmarks by size, position, and rotation (Claude, 2008).

As a prerequisite for OA, we converted all pictures to pure black and white to facilitate the extraction of the (*x*; *y*) coordinates of the fish outlines (Bonhomme et al., 2014; Figure S3a–b). Images were fully desaturated to black and white using GIMP 2.8.22 (https://www.gimp.org/). Additionally, we identified five landmarks on the outlines in order to remove differences in image rotation and size (Caillon et al., 2018; Figure S3). The coordinates of the outlines were standardized using a Procrustes superimposition on these five landmarks. Elliptic Fourier transforms (EFT) decomposed separately the *x* and *y* coordinates of the outlines into a harmonic sum of trigonometric functions, weighted by coefficients that can further be used as quantitative variables (Figure S3d–g). EFT is a curve fitting technique that describes the outline of an object by summing multiple trigonometric (sine and cosine) functions. This allows the description of the whole outline, not being limited to a restricted number of landmarks. The more functions are incorporated, the more precise the shape outline is defined. More details can be found in (Bonhomme et al., 2014; Claude, 2008) and a tutorial explaining OA on fish communities is available online (Caillon et al., 2018): https://rfrelat.github.io/FishMorpho.html. We retained 15 harmonic coefficients required to obtain 99% of the cumulative harmonic power (Bonhomme et al., 2014).

Given our focus on adult fishes, it is unlikely that allometry influenced any of the morphometric approaches used here. Further, all methods capture size‐standardized morphology by default. TM uses unitless ratios between measures, whereas GM and OA use standardized coordinates. Therefore, no allometric correction procedures were applied.

### 2.3 Defining and comparing morphospaces

To characterize the morphological variability among nominally herbivorous fish, we ordered individuals according to traits, landmarks, and outlines in three separate Principal Component Analyses based on Euclidean distance (i.e., morphospaces). For an even comparison between morphospaces, we retained the first three principal components (PCs) in all cases. This was justified by the distribution of eigenvalues (i.e., additional explained variance by successive PCs) for TM and OA, and was more than sufficient for LA, for which two PCs were optimal.

The loadings of the traits, landmarks, and harmonic coefficients describing the outlines were used to interpret the PCs, describing the main dimensions of morphological variability identified by each approach. For LA and OA, we used the loadings to reconstruct the configuration of landmarks and the outlines along PCs to aid visual interpretation. We extracted the scores of individual fish on PC1‐PC3 and quantified the Pearson correlation coefficients in order to compare the topology of all three morphospaces.

The degree of differentiation among taxonomic and feeding functional groups within the morphospaces is informative of the potential correlation (not causation) between morphology and taxonomy, and morphology and feeding category, respectively. Within the morphospaces derived from each approach, we assessed the degree of differentiation among (a) Acanthuridae, Labridae (Scarinae), and Siganidae, (b) 11 genera, and (c) 10 feeding functional groups. To quantify the degree of differentiation among both taxonomic and feeding functional groups, we computed the silhouette values (*s*) per group, which indicate how distinctly clustered these groups are. *s* is based on the difference between the average distance among individuals within a group (tightness) and the average distance between each individual within a group and each individual within neighboring groups (separation; Rousseeuw, 1987). In distinct groups, the within‐cluster distances are smaller than the separation. *s* ranges from −1 (lowest degree of clustering) to +1 (highest degree of clustering), is sensitive to the number of groups, and lacks information about the significance of these groups. Therefore, we tested the significance of the grouping using a null model approach. We randomly assigned the taxonomic or feeding group to each species and calculated *s* in these shuffled groups. We repeated this random shuffling of taxonomic or feeding group 1000 times. The *p*‐value was estimated by comparing the observed values to the values obtained from the 1000 iterations of the null model. We then adjusted the *p*‐value to account for multiple testing following the ‘false detection rate’ method (Benjamini & Yekutieli, 2001).

### 2.4 Detecting spatial patterns in morphological diversity

We tested whether the choice of morphometric approach influenced inferred spatial patterns of morphological diversity of fish assemblages. To this aim, we combined the morphospace obtained with TM, LA, and OA, with in‐situ estimates of species occurrence (i.e., presence–absence) recorded by unmanned stationary video cameras on 12 forereefs in Palau (Bejarano et al., 2017). We consider this study a useful and accessible example for our comparative aims, yet recognize that others encompassing larger gradients (e.g., Johnson et al., 2019) are more inclusive of the full spectrum of species’ trait–environment associations. We first averaged individuals’ scores to obtain the three morphospaces aggregated per species. For each site and within each morphospace, we then computed two indices of morphological diversity, namely richness, and dispersion (Laliberté & Legendre, 2010). Richness is defined as the percentage of morphospace volume filled by a community (Laliberté & Legendre, 2010), thus it represents here the range of morphological variability spanned by the recorded fish assemblages. Dispersion is the mean distance of all species within a community from their center of gravity (Laliberté & Legendre, 2010). It is therefore interpreted here as the average deviation of the observed assemblage from their “average” morphology. We tested whether the choice of morphometric approach changed the ranking order of sites according to morphological diversity of their fish assemblages. The indices derived from each approach were compared using a Spearman rank‐correlation test.

To compare morphological richness and dispersion derived from the three different approaches (i.e., TM, LA, and OA) across levels of wave exposure, we used analysis of variance (ANOVA). We ran one ANOVA per metric per approach, verifying that in all cases the response variable was normally distributed (via Shapiro–Wilk's tests) and the homoscedasticity assumption was met (by plotting the models’ residuals against the fitted values; Zuur et al., 2010). ANOVAs indicating a significant effect of wave exposure were followed by a Tukey test to test for pairwise differences. Differences were considered significant based on a Bonferroni‐corrected threshold considering the number of tests.

All statistical analyses were conducted in the programming environment R 3.6 (R Core Team, 2019). Morphometric analyses were conducted with the package Momocs 1.2.9 (Bonhomme et al., 2014), the morphological diversity indices were calculated with the package FD 1.0 (Oksanen et al., 2017), and the clustering coefficients with the package cluster 2.1 (Maechler et al., 2019). Additionally, we provide the script and dataset on GitHub, together with a tutorial explaining the different steps to apply and compare the three morphometrics approaches (https://rfrelat.github.io/CoralFishes; see Data accessibility statement).

## 3 RESULTS

### 3.1 Morphological variation among individuals

**FIGURE 1:**
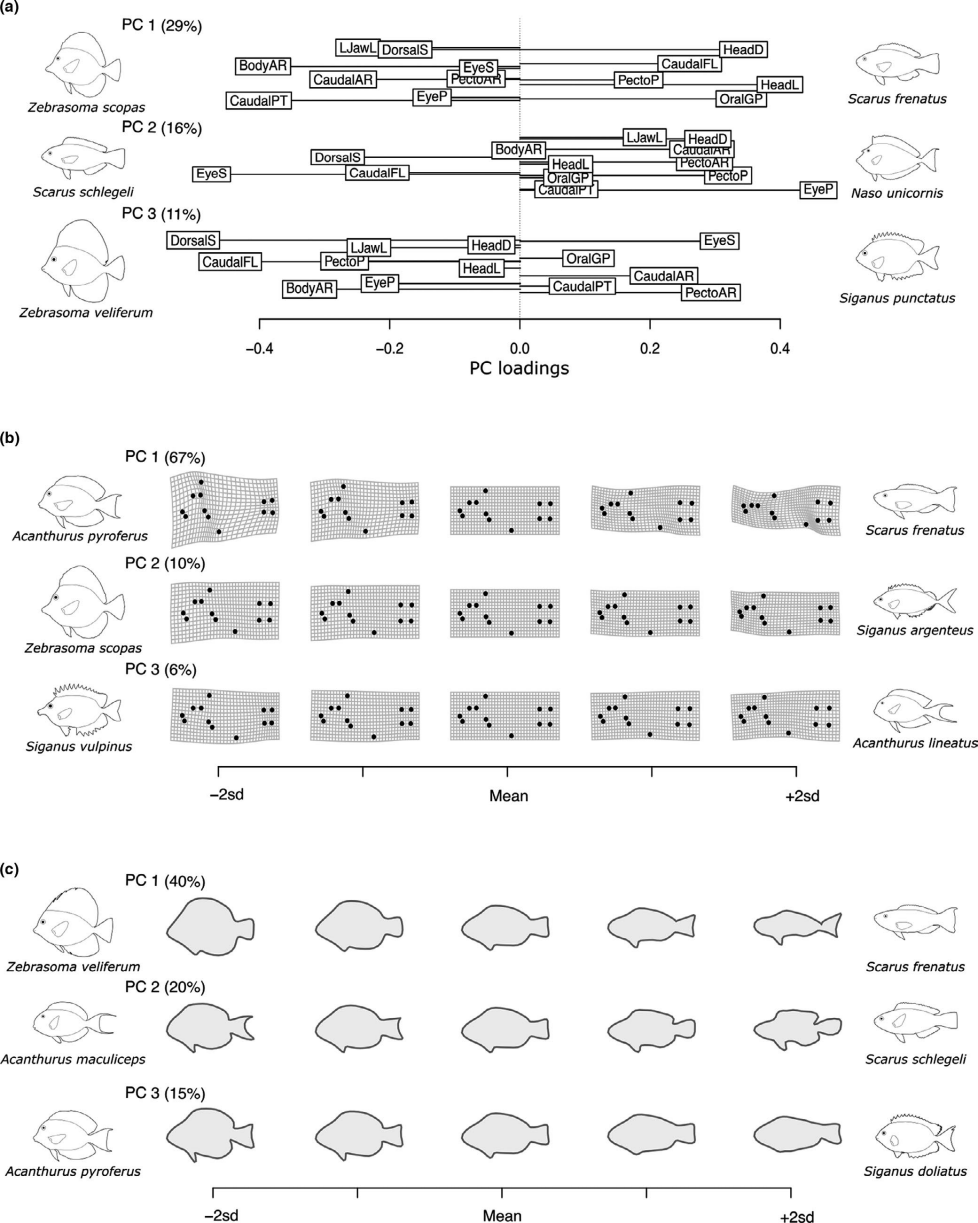
Diagram indicating the main dimensions of morphological variation across individual fish in terms of traits, landmarks, and outlines. (a) Loadings of the morphological traits on the first three PCs of TM. Trait abbreviations are explained in Table S4. (b) Reconstructed position of the landmarks along the first three PCs of LA. (c) Reconstructed outline along the first three PCs of OA. The percentages indicate the additional variability explained by each PC. Outlines of species with extreme scores on the PCs are represented at opposite sides of the axes

In the morphospace derived from TM, the first three PCs captured, respectively, 29%, 16%, and 11% of the variability among individuals as described by 13 morphological traits (Figure 1a). The trait loadings on PC1_TM_ indicated a morphological gradient from disk‐shaped bodies with high values of caudal peduncle throttling (i.e., slender caudal peduncles connected to deep caudal fins equivalent to high thrust efficiency) towards elongated bodies with large heads. At opposite ends of this spectrum, we found disk‐shaped *Zebrasoma scopas*, which has a small head and narrow caudal peduncle, and *Scarus frenatus* with its elongated fusiform body, large head, and broad caudal peduncle. PC2_TM_ marked a continuum between fish with large eyes located low in the vertical axis of the head and ventrally positioned pectoral fins (e.g., *S.* *schlegeli*) and fishes with smaller eyes positioned high in the vertical axis of the head and dorsally positioned pectoral fins (e.g., *Naso unicornis*; Figure 1a). Lastly, PC3_TM_ denoted differences in fin features and eye size (Figure 1a). These differences are observed, for example, in *Z. veliferum*, which has a larger dorsal spine, more elongated pectoral fins, and smaller eyes than *Siganus punctatus*.

Using LA, the first three PCs captured, respectively, 67%, 10%, and 6% of the variability among individuals as described by 12 landmarks. PC1_LA_ was related to differences in elongation, head depth, and caudal peduncle shape (Figure 1b). *Acanthurus pyroferus*, for instance, had more widely spread head landmarks, a more anterior anal fin, and less‐spread‐out caudal peduncle landmarks compared to *S.* *frenatus*. Individuals segregated along PC2_LA_ according to their eye‐to‐mouth distance, pectoral‐to‐anal fin distance, and caudal peduncle shape (Figure 1b). For example, *Z*. *scopas* had larger eye‐to‐mouth distances and shorter pectoral‐to‐anal fin distances compared to *S*. *argenteus*. Lastly, the spread of individuals along PC3_LA_ was related to their differences in the location of caudal peduncle landmarks, relative pectoral fin position, and eye size. At one end of PC3_LA_, *Siganus vulpinus* had shorter and narrower caudal peduncles, pectoral fin insertion points lower than the mouth level, and larger eyes compared to *Acanthurus lineatus* found at the opposite end of PC3_LA_ (Figure 1b). These patterns were evident through visual inspection of Figure 1 and confirmed by the comparison of the loadings of the landmarks on PC2_LA_ and PC3_LA_ (e.g., the loading of the *x*‐coordinate of the pectoral fin had an opposite sign to the loading of the *x*‐coordinate of the anal fin on PC2_LA_).

Using OA, the first three PCs captured, respectively, 40%, 20%, and 15% of the variability among individuals in their harmonic coefficients. PC1_OA_ denoted variability in body elongation and anal fin position (Figure 1c) and highlighted the difference between disk‐shaped *Z*. *veliferum* with anal and pelvic fins closer together compared to elongated *S*. *frenatus* which widely separated anal and pelvic fins. PC2_OA_ highlighted variations in the outline of caudal fins and heads, including the position of the mouth. *A*. *maculiceps*, for instance, had a deeply lunate caudal fin, more rounded head, and a more ventrally positioned mouth compared to *S*. *schlegeli* which is characterized by a slightly convex caudal fin and pointed head (Figure 1c). Lastly, PC3_OA_ marked a range between fishes with large (e.g., *A*. *pyroferus*) and small (e.g., *S*. *doliatus*) fins, especially noticeable in the pelvic fins (Figure 1c).

### 3.2 Comparison among morphospaces

**FIGURE 2:**
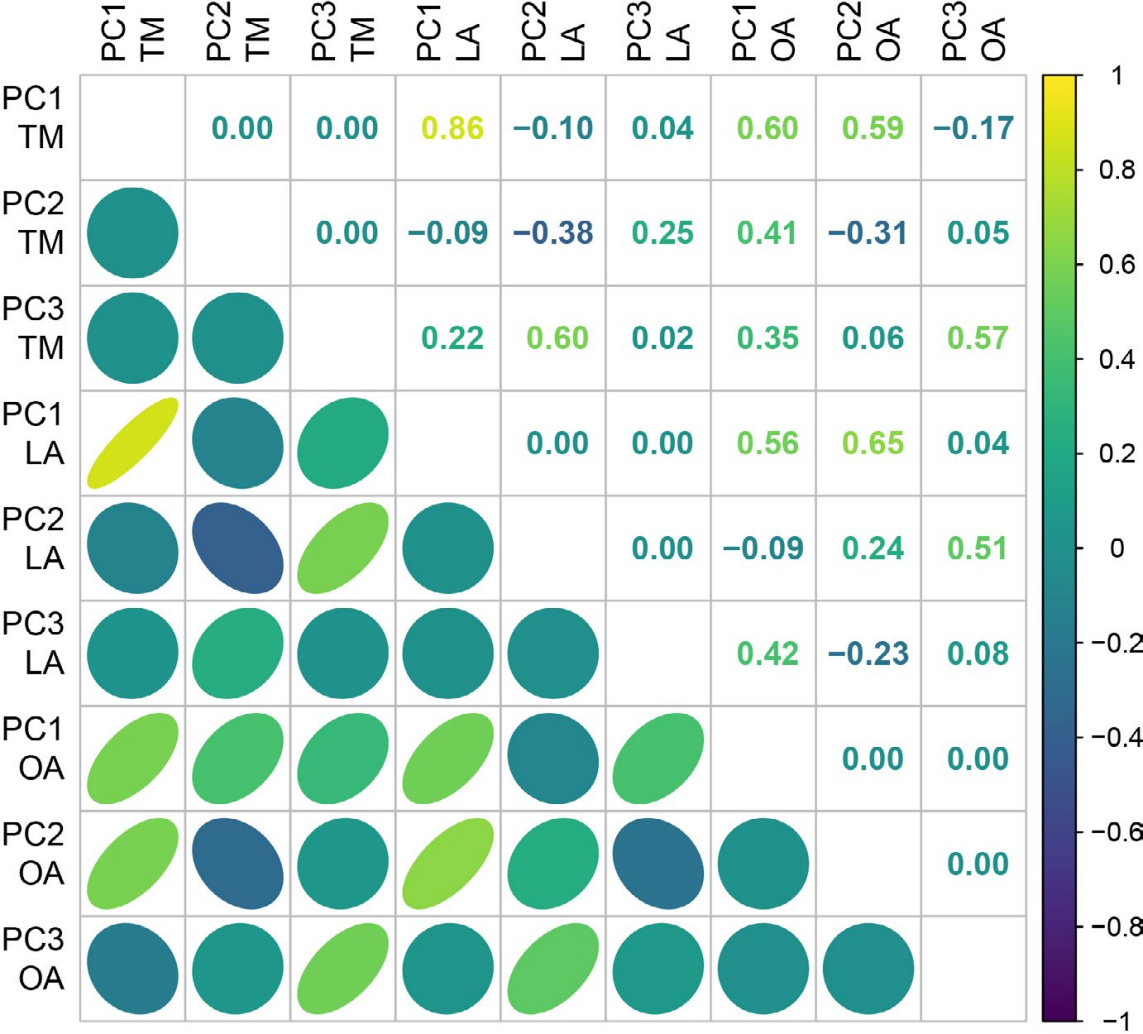
Grid diagram showing the pairwise Pearson correlation coefficients (top right) between the individual scores on the first three PCs obtained from the different methods (TM, LA, OA). Ellipses (bottom left) represent the distribution of the observations. Colors indicate the direction of the correlation (i.e., yellow for positive and blue for negative). Both the shape and color intensity of the ellipses reflects the value of the Pearson correlation coefficients (i.e., narrowest and darkest ellipses correspond to the highest correlation coefficients, whereas widest and lightest ellipses mark the lowest correlation coefficients)

While quantifying different aspects of fish morphology, all morphometrics approaches identified similar main dimensions of morphological variation. Individuals’ scores on PC1_TM_ were strongly and positively correlated to individuals’ scores on PC1_LA_ (Pearson correlation coefficient *r* = .86, Figure 2), and both of these were also positively correlated with individuals’ scores on PC1_OA_ and PC2_OA_ (*r* > .50, Figure 2). This implies that individuals consistently distributed along a main axis of variation ranging from disk‐shaped to elongated bodies across the three approaches as used here. Interestingly, using OA, both PC1 and PC2 captured information on fish elongation (Figure 2).

Individual scores on PC2_TM_ were negatively yet weakly correlated to those on PC2_LA_ and PC2_OA_ (*r* > −.30, Figure 2). This indicates that the inherent complementarity of the three approaches as applied here lies mainly on the secondary axis of morphological variability they capture. PC2_TM_ identifies differences in eye size and pectoral fin position, PC2_LA_ detects differences in eye‐to‐mouth distance, and PC2_OA_ captures differences in the outlines of the caudal fin and head.

Furthermore, PC3_TM_ and PC2_LA_ were moderately and positively correlated with each other and with PC3_OA_ (*r* > .50, Figure 2). These PCs denoted differences in the fins’ sizes and width of the caudal peduncle among individuals. Additionally, PC3_TM_ and PC3_OA_ were linked to differences in dorsal fin shape while PC3_TM_ and PC2_LA_ were linked to differences in pectoral fin aspect ratio and position, respectively.

### 3.3 Differentiation of taxonomic and feeding functional groups

**FIGURE 3:**
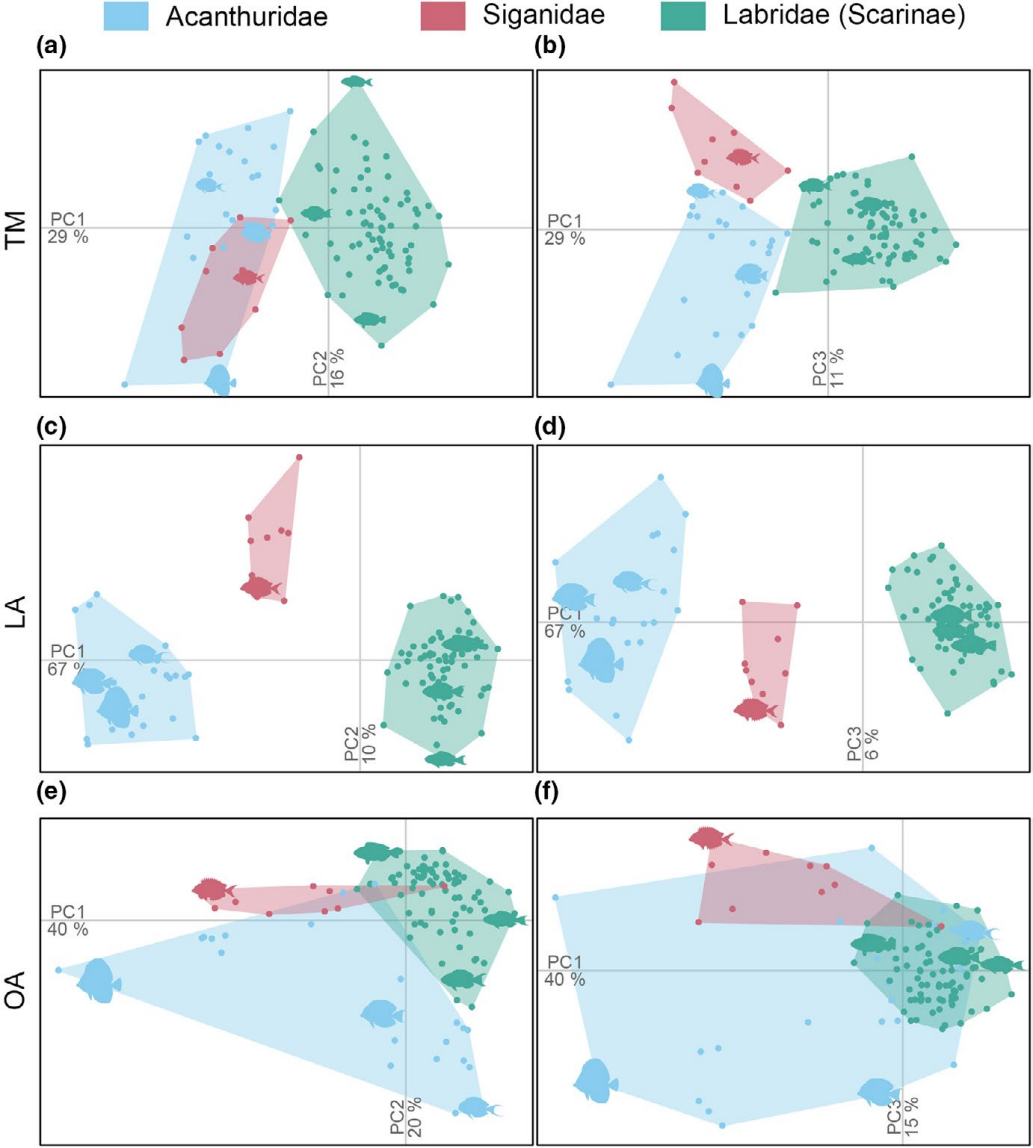
Dissimilarity among Acanthuridae, Siganidae, and Labridae (Scarinae) within morphospaces defined by traits using TM (a–b), landmarks using LA (c–d), and outlines using OA (e–f). Dots are individual fish ordered on PCs 1 and 2 (a, c, e) and PCs 1 and 3 (b, d, f), color‐coded and enclosed in separate convex hulls per taxonomic group. Seven images with distinct shapes are represented in all morphospaces

The elongation gradient captured by PC1_TM_, PC1_LA_, PC1_OA_, and PC2_OA_ marked a continuum from surgeonfishes (Acanthuridae) to rabbitfishes (Siganidae) to parrotfishes (Labridae, Scarinae; Figure 3). These groups were more different from each other in their landmarks (*s* = 0.66) than in their traits or outlines (*s* = 0.45 and 0.43, respectively, Table 2; Figure 3). However, all three approaches led to significant morphological differentiation among families, as indicated by a higher *s* than expected by chance based on a null‐model with 1000 iterations (adjusted *p*‐value < .01; Table 2).

**TABLE 2:** Morphological differentiation among taxonomic and feeding groups measured by silhouette values

Approach	TM	LA	OA
***Taxonomic groups***			
Acanthuridae, Labridae (Scarinae), Siganidae	0.45 (0.00)*	0.66 (0.00)*	0.43 (0.00)*
*Acanthurus*, *Bolbometopon*, *Calotomus*, *Cetoscarus*, *Chlorurus*, *Ctenochaetus*, *Hipposcarus*, *Naso*, *Scarus*, *Siganus*, *Zebrasoma*	−0.03 (0.00)*	0.05 (0.00)*	−0.13 (0.00)*
**Interaction with calcareous reef matrix** (Bioerosion; excavators, scrapers, non‐bioeroders)	0.12 (0.00)*	0.27 (0.00)*	0.01 (0.17)
**Interaction with algal turfs** (Algal turf removal; intensive farmers, croppers, removers, non‐consumers)	0.14 (0.00)*	0.42 (0.00)*	0.10 (0.00)*
**Interaction with upright macroalgae** (macroalgal browsers, incidental consumers, non‐consumers)	−0.14 (0.89)	−0.17 (0.78)	−0.19 (0.90)

Note

Significance is indicated by asterisks and is based on *p*‐values (in parentheses) estimated from a null model with 1000 repetitions and adjusted for multiple testing.

The morphological differentiation among genera was less marked than the separation among families, yet significant (Table 2; Figure S4). Genera were more different from each other in their landmarks (*s* = 0.05) than in traits or outlines (*s* = −0.03 and −0.13, respectively; Table 2; Figure S4). Interestingly, the genus *Zebrasoma* was different from all other Acanthuridae only when using OA (Figures S4–S5). Specifically, *Zebrasoma* had truncate, emarginate, or rounded caudal fins, different from the lunate caudal fins of other Acanthuridae.

**FIGURE 4:**
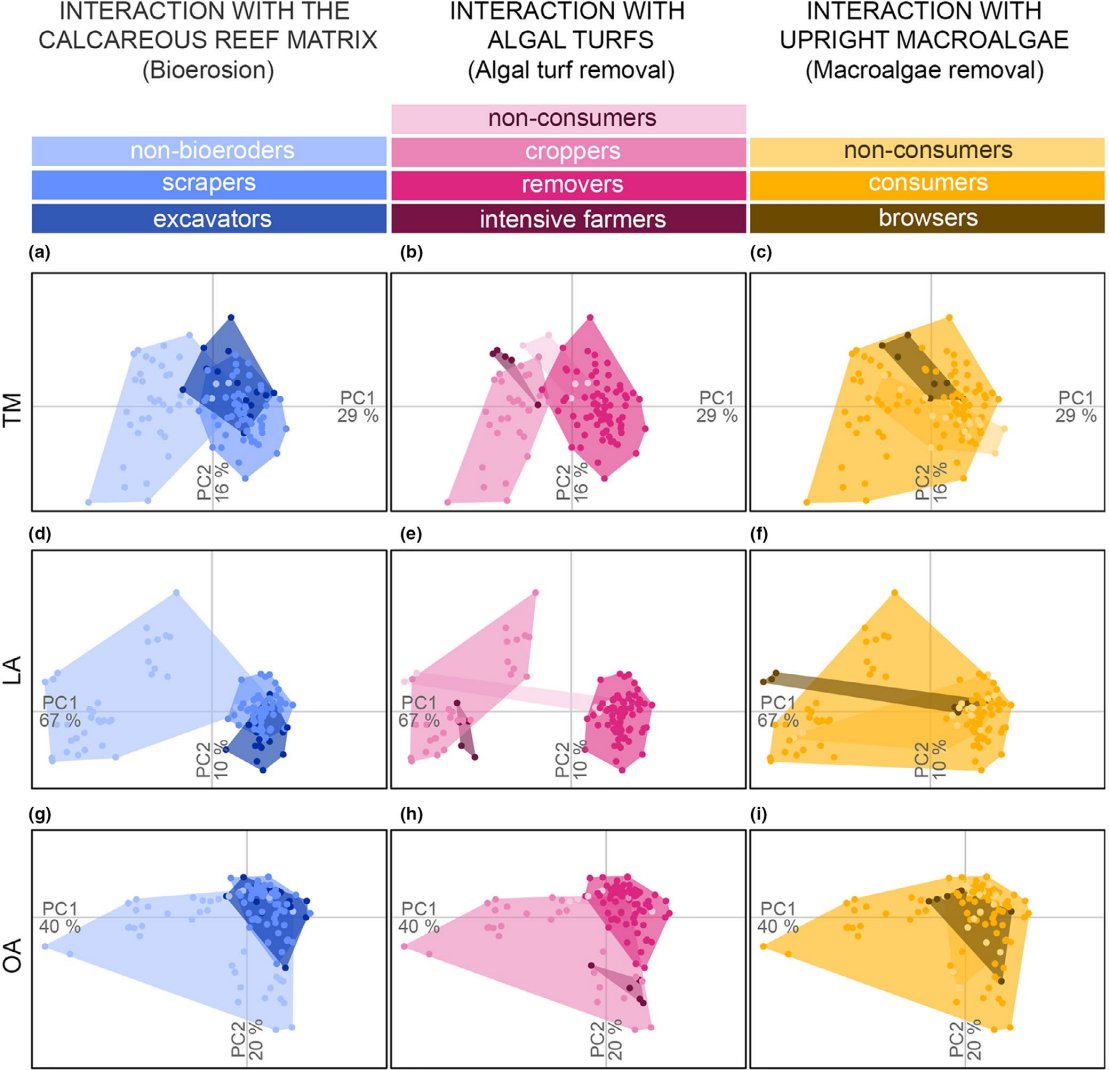
Dissimilarity among feeding functional groups (Table 1) within morphospaces defined by traits using TM (a–d), landmarks using LA (e–h), and outlines using OA (i–l). Dots are individual fish ordered on PCs 1 and 2, color‐coded and enclosed in convex hulls by feeding functional group. Feeding functional groups enclose species known to interact differently with (i) the calcareous reef matrix, (ii) algal turfs, and (iii) upright macroalgae, and thus contribute differently to bioerosion (i.e., non‐bioeroders, scrapers, and excavators), algal turf removal (i.e., non‐consumers, algal turf removers, croppers, and farmers), and macroalgal removal (i.e., non‐consumers, incidental consumers, and macroalgal browsers; Bejarano et al., 2019, Table 1)

Predefined feeding functional groups that contribute differently to macroalgal removal (Bejarano et al., 2019) were morphologically undifferentiated from each other regardless of the approach used (Table 2; Figure 4c,f,i). Feeding functional groups that contribute differently to algal turf removal were marked as also morphologically distinct from each other by all morphometric approaches (Table 2; Figure 4b,e,h). Feeding functional groups that contribute differently to bioerosion differed also in their traits and landmarks but not in outlines (Table 2; Figure 4a,d,g). The cohesion within certain groups and their disparity from others varied depending on the morphometric approach used (Table 2; Figure 4). Fishes that excavate the calcareous reef matrix, and fishes that remove algal turfs, for instance, comprised more cohesive groups of individuals when using LA and OA than TM. Using landmarks, excavators were morphologically distinct from non‐bioeroders and algal turf removers were morphologically distinct from algal turf croppers. These differences were less pronounced when using traits or outlines (Table 2; Figure 4b,e,h).

### 3.4 Spatial patterns of morphological diversity

**FIGURE 5:**
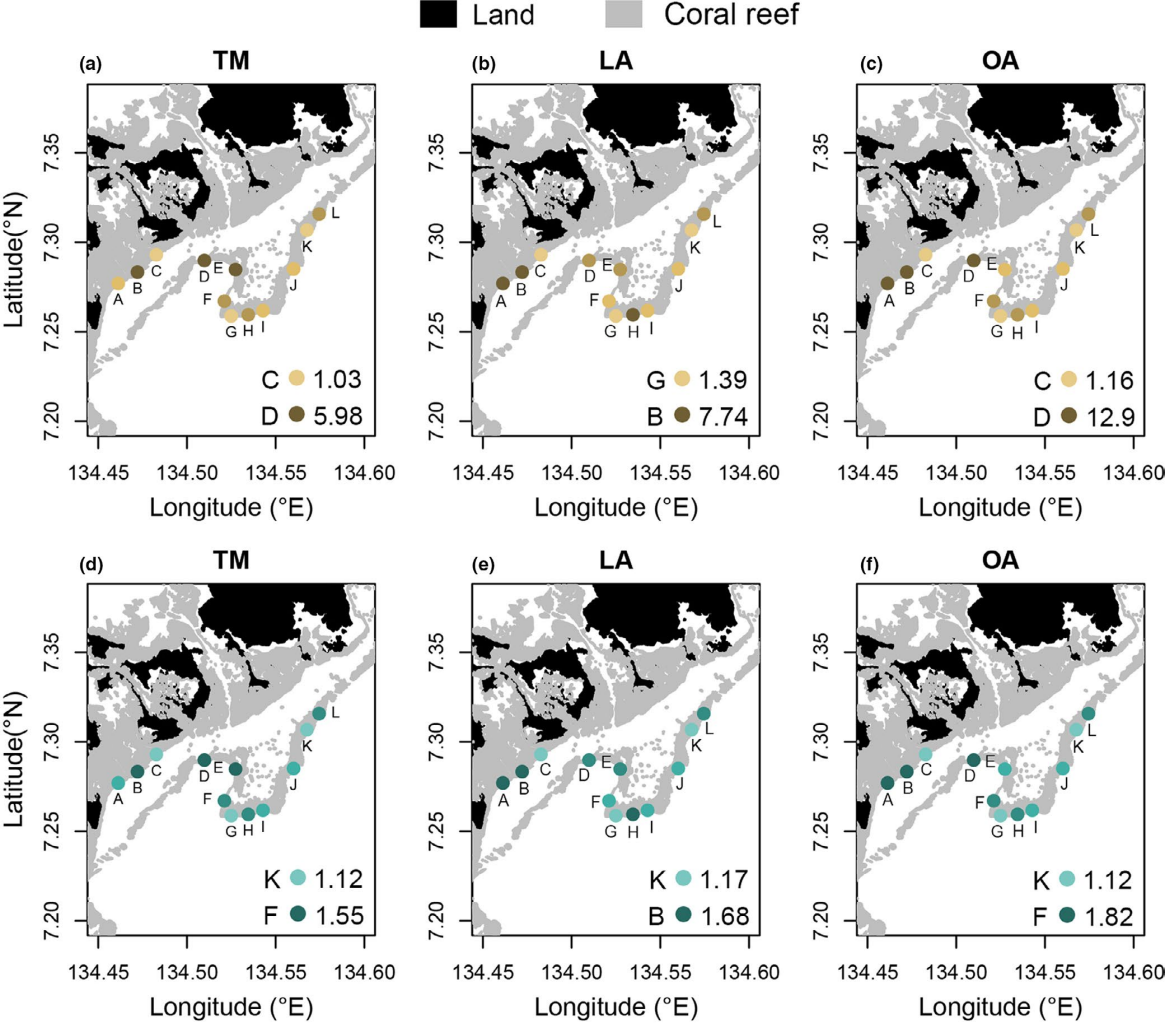
Reef sites ranked according to herbivore morphological diversity. Sites as dots in maps of Ngederrak and Uchelbeluu reefs (Palau), ranked according to herbivore morphological diversity based on morphological datasets and presence‐absence of nominally herbivorous fish recorded in situ using stationary video cameras. Sites A‐L are color‐coded differently per map to indicate their rank position from highest to lowest in (a–c) morphological richness and (d–f) dispersion when using TM, LA, and OA. The highest and lowest values with the corresponding sites are also indicated in the bottom right corner of each map

**FIGURE 6:**
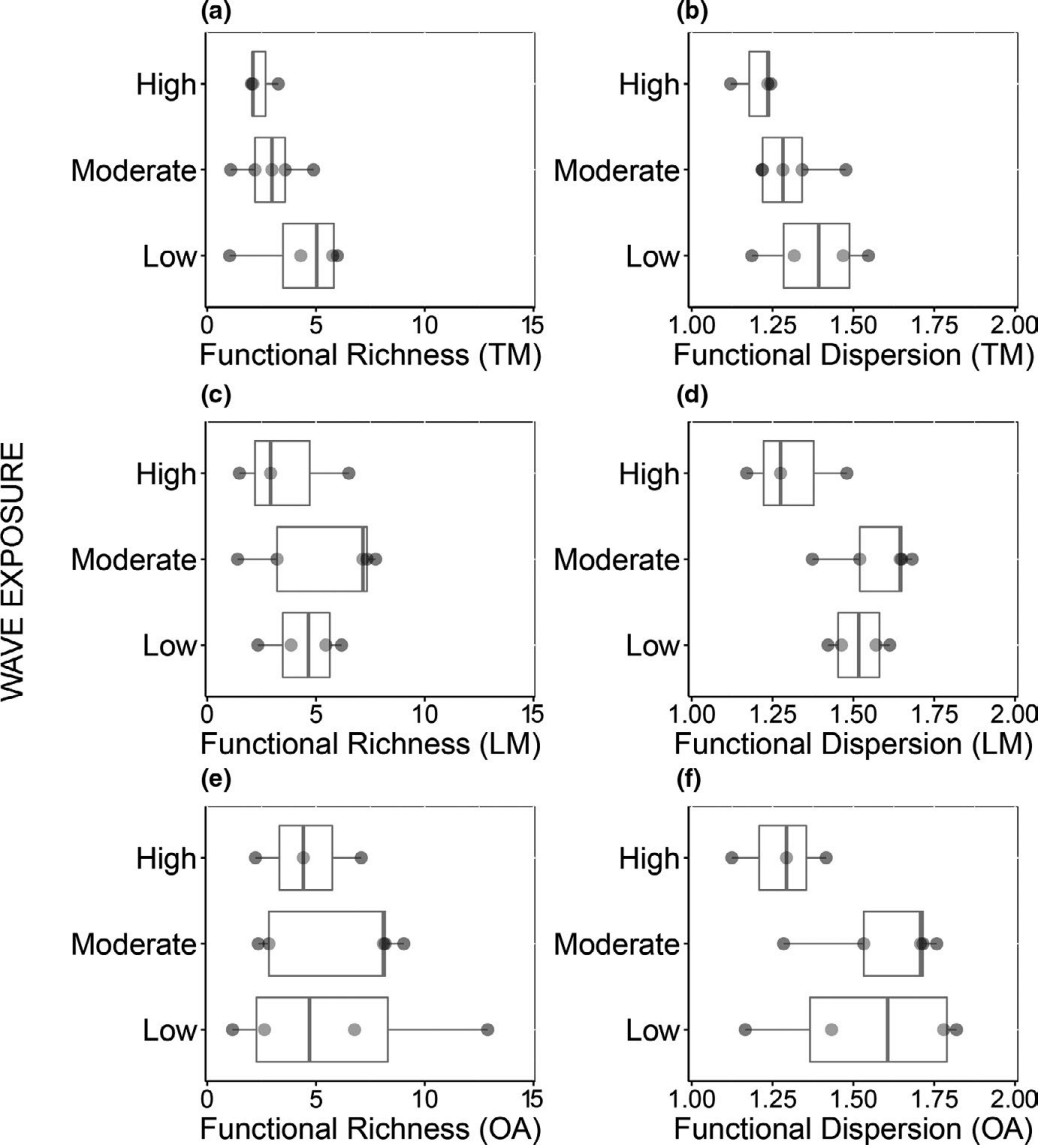
Herbivore morphological diversity across wave exposure levels. Mean (±SE) morphological richness (a, c, e) and morphological dispersion (b, d, f) derived using traits (TM), landmarks (LM), and outlines (OA) in reef areas subject to low, moderate, and high wave exposure on Ngederrak and Uchelbeluu reefs (Palau)

When ranked according to the morphological richness and dispersion of their fish assemblages, the exact position of some of the 12 reef sites differed depending on the morphometric approach used (Figure 5). The overall rankings were, however, highly correlated between pairs of approaches (Rho > 0.70, *p*‐values < .05). Site D, for instance, ranked highest in morphological richness among all sites when using TM and OA, whereas it ranked sixth when using LA (Figure 5). Site E ranked within at least four positions of difference in morphological richness across methods (Figure 5). Relatively broader spatial patterns were conserved across approaches. Site B, for example, ranked consistently within the top three in morphological richness, whereas sites C, G, and K were within the bottom three in morphological richness, and I and K within the bottom three in morphological dispersion (Figure 5). Furthermore, regardless of the morphometric approach used, no significant differences were found in morphological richness or morphological dispersion among wave exposure levels (Figure 6; Table S4).

## 4 DISCUSSION

We characterized the variability among nominally herbivorous fish in three different aspects of their morphology, namely traits (TM), landmarks (LA), and outlines (OA). By their very nature, all three methods highlight different aspects of morphological diversity (e.g., OA cannot capture pectoral fin features), thus are complementary approaches to study morphology. Nevertheless, all three methods indicated that individuals were most variable in the extent of body elongation. Each approach captured different secondary dimensions of morphological variability among individuals (e.g., eye size and position, eye‐to‐mouth distance, and caudal fin outline). Importantly, morphological differences among families, genera, and feeding groups were more pronounced when examining their landmarks than when considering traits or outlines. This implies that the choice of morphometrics approach may lead to different interpretations regarding the morphological disparity among taxonomic and functional groups. Combining each of the three different morphological datasets with species presence‐absence data collected in a relatively small number of reef sites (*n* = 12) distributed across a strong wave exposure gradient, we computed morphological diversity of fish assemblages. We conclude that the different methods led to different estimates of the sites with the highest and lowest morphological diversity, indicating that the nature of the morphometric method leads to an emphasis on different aspects of morphology. Despite these differences, conclusions regarding the relationship between wave exposure and morphological richness were robust to the morphometric approach used. Whether our conclusions hold true when considering a more comprehensive species pool, a larger number of sites, or sites distributed over longer or weaker environmental gradients, remains to be tested. Prioritizing species richness hotspots is one of the most common strategies to conserve biodiversity (Norman & White, 2019). The location of such hotspots may, however, be elusive and context‐dependent given that biodiversity is multifaceted and the different facets are not necessarily spatially congruent (Doxa et al., 2020; Tolimieri et al., 2015). The integration of more than one biodiversity metric or a more informed use of the different metrics depending on the particular conservation goals, has been called for (Cadotte & Tucker, 2018; Devictor et al., 2010a; Tolimieri et al., 2015). In the same vein, our results caution against labeling particular sites as morphological diversity hotspots when metrics consider any single aspect of morphology (e.g., traits) because the numerical values of morphological diversity are subject to the chosen method, that is, to which aspect of morphology is the emphasis given.

Body elongation was consistently identified as the main axis of morphological variation among nominally herbivorous fishes. Elongated parrotfish separated clearly from disk‐shaped surgeonfish. This result is coherent with a study using LA, which identified elongation as the main variability axis among 3000 tropical reef fishes (Claverie & Wainwright, 2014a). A recent study using TM also confirmed the importance of elongation across more than 6000 teleost fishes (Price et al., 2019). More generally, elongation was suggested to be the main variation of body shape among vertebrates (Collar et al., 2013). In fish, elongation is known to correlate with activity, responses to fluctuations in environmental factors, and fish metabolic rate (Bejarano et al., 2017; Claverie & Wainwright, 2014a). This does not imply, however, that elongation reliably tracks metabolic rate and activity, as some deep‐bodied taxa are highly active (Wegner et al., 2015). Although they quantified different aspects of fish morphology, all morphometrics approaches identified similar main dimensions of morphological variation. To a large extent, this may be driven by phylogenetic signal (i.e., phylogenetic conservatism of both morphology and trophic role; Blomberg et al., 2003; Harvey & Pagel, 1991; Pavoine et al., 2013; Wainwright, 2007). Arguably, using phylogenetic PCA (*p*PCA) could clarify this. However, *p*PCA would constrain the PCAs based on phylogenetic distance, and all morphospaces would thus contain both morphological and phylogenetic signals, which would complicate the identification of differences among approaches in capturing morphology. Therefore, classic PCA remains a better fit to the aims of our study.

Prior comparisons among morphometrics approaches considered TM versus LA (applied to human molars, moths, and lizards) and revealed the complementarity between these methods. In general, LA contributed important morphological information otherwise undetectable when using TM, and these methods led to different interpretations regarding the similarities among the measured items (Bernal, 2007; Fabre et al., 2014; Mutanen & Pretorius, 2007). Our study extended the scope of prior comparisons to span TM, LA, and OA. Applied to a multispecies assemblage, we find that the three approaches differed in the detection of variation in the morphology of fins and head shape. For instance, OA captured large variability in caudal fin shape among Acanthuridae and separated *Zebrasoma* from *Acanthurus*. These groups were indistinguishable in terms of traits in TM and landmarks in LA. Differences in caudal fin shape are one of the main determinants of differences in various maneuvering functions (Xiong & Liu, 2019). Forked or semilunate caudal fins, for instance, contribute to reduce drag, which is conducive to cruising, whereas trapezoidal caudal fins are well‐adapted for accelerating, and fanned fins are suitable for maneuvering (Krishnadas et al., 2018; Webb, 1988). It remains uncertain why and how the Acanthuridae diverged more broadly than other groups in caudal fin shape. Dorsal fins also play an important role in fish maneuverability. In particular, dorsal fin size, described by TM and OA but not captured by LA, influences fish stability and the capacity for sudden turns (Drucker & Lauder, 2005). Small dorsal fins distinguished the Siganidae from both Acanthuridae and Scarinae. The Siganidae were also characterized by different pectoral fin shape and position, known to correlate with swimming speed (Bellwood et al., 2002; Dumay et al., 2004; Watson & Balon, 1984). Differences in pectoral fin shape and position, respectively, captured by TM and LA, are inherently undetected by OA, thus highlighting the complementarity among methods. Individual fishes differed also in head length and depth, as well as in eye and mouth position. The three approaches identified the link between long heads and elongated bodies (e.g., *S. frenatus*), and between deep heads and disk‐shaped bodies. Other key characteristics of the head are the position and size of the eye, which can only be captured by TM and LA. The eye position gives important insight into locomotion and vertical position in the water column (Gatz, 1979; Villéger et al., 2010), while the eye size correlates with diet and prey detection (Boyle & Horn, 2006; Villéger et al., 2010).

The discrepancies captured here among the morphometrics methods reflect primarily that, by nature, each method measures different aspects of fish morphology. TM, for instance, is fundamentally different from LA because it is based on vectors corresponding to fish body parts, which are often interpreted in a functional ecology context. Meanwhile, LA produces an *n*‐dimensional array of coordinates that forms a single configuration (i.e., shape). It is therefore likely that TM and LA result into relatively different interpretations of fish morphology. In addition, differences between TM and LA are also influenced by the choices of traits and landmarks included in the analysis. In our study, the selection of traits and landmarks was not arbitrary but guided by the sets most commonly used to study fish morphology using TM and LA (Bellwood et al., 2014; Claverie & Wainwright, 2014a; Su et al., 2019; Villéger et al., 2010). For example, with TM, head length and oral gape position (which score highly on PC1, Figure 1), are not captured by any of the landmarks selected for LA, and inherently missed by OA. Similarly, one of the primary sources of variation across individuals using LA is the insertion point of the anal fin, yet none of the TM traits used here and usually measured in other studies (i.e., related to locomotion and feeding) capture this feature. To test for the sensitivity of our results to the choice of traits and landmarks, ancillary analyses were conducted on subsets of more overlapping traits and landmarks (Table S5). Using these subsets caused no major change in our conclusions (Figures S6 and S7; Tables S6 and S7).

Furthermore, our results indicate that, given the sets of traits and landmarks selected here, OA is the only one of the investigated approaches that captures variation in caudal fin shape. This is, however, an artifact of our choice of traits and landmarks, which followed common practice in published literature. If caudal fin shape had been of interest for LA studies, it could have been included, for instance, by the use of sliding semi‐landmarks. Similarly, TM could have included linear measurements capturing caudal fin shape traits (e.g., depth of the caudal fin) rather than using only caudal fin area. This points at the important conclusion that the use of any of these morphometrics approaches does not establish absolute or fixed similarities or differences among species. Rather, each method provides insights into different aspects of morphological diversity, thereby showing the complementarity of the different approaches. This complementarity provides valuable opportunities for conscious consideration of the anatomical aspects of interest given the objectives stated in animal morphology and diversity studies. The choice of method may, however, be restricted for purposes involving the reconstruction of ancestral morphology and thus relying on specimens with incompletely preserved body parts (e.g., fossils from museum collections, Siqueira et al., 2019). Morphology‐ecosystem function associations are a cornerstone of evolutionary biology (Irschick, 2002). Strong linkages have been found between either particular morphological traits or body plans inferred from a set of traits, and (a) the performance of ecological tasks and thus fitness (Wainwright, 1988), (b) diet and degree of dietary specialization (Brandl et al., 2015; Frédérich & Adriaens et al., 2008, Frédérich & Arnaud et al., 2008), and (c) contrasting social behaviors (Brandl & Bellwood, 2013). Studies investigating the relationship between ecosystem function and morphology as described by attributes other than traits (e.g., body landmarks or outlines) are less common. This may be partially because connections between traits and functions are better understood than are those between functions and landmarks or outlines (Villéger et al., 2017), or because associations between landmarks or outlines and functions might be weaker than associations between functions and the traits themselves. Focusing on a highly diverse group of reef fishes, our study quantifies the morphological disparity among groups of species that contribute differently to bioerosion, algal turf removal, and macroalgae removal in terms of traits, landmarks, and outlines. We did not aim to establish a causal link between form and function. This would require establishing first a relationship between organism morphology and performance, and then testing whether performance drives resource use (Sibbing & Nagelkerke, 2001; Wainwright, 1991). Testing whether the location of organisms in morphospaces predicts their trophic function, or tracing evolutionary trajectories of herbivorous reef fish (e.g., using random forest models, Pigot et al., 2020) also laid outside the scope of this study. Rather, we concentrate on replicating a practice engraved in ecological studies for decades (i.e., using functional groups that utilize certain aspects of the environment in similar ways Steneck & Watling, 1982). In our case, functional groups were pre‐defined (independently of morphology) by Bejarano et al. (2019; Table 1) and here we provide a first assessment of the morphological disparity within and among these groups.

In our case, only feeding functional groups that interact differently with algal turfs, and therefore contribute differently to algal turf removal, were consistently separated from one another by all morphometric approaches. These groups where, however, most different from each other in their head and tail landmarks. Specifically, algal turf removers (i.e., parrotfishes) tend to have bullet‐shaped heads where landmarks concentrate closer together, whereas croppers have deeper heads with more widely spaced landmarks. This difference in morphology is less noticeable using outlines, at least partially due to the high intra‐group variability in the contours of algal turf croppers (Figure 4h). Algal turf removers formed a particularly cohesive group based on landmarks and outlines, indicating that LM and OA missed the interspecific variability in parrotfish shape (e.g., eye size and position, head length, and caudal peduncle throttling). The examination of the form‐function relationships may lead to the identification of ecomorphotypes with a certain level of interspecific cohesion or spread (Mihalitsis & Bellwood, 2019). Our findings demonstrate that considering aspects of morphology other than traits (e.g., landmarks) may affect the cohesion of these groups. Some morphological differences among feeding functional groups were noticeable only when examining landmarks. Whether the potential associations highlighted here between morphology and feeding functional groups are causally linked remains to be tested and requires careful demonstration of the linkages between form‐performance and resource use.

Spatially referenced biodiversity metrics are often integrated with data on connectivity, thermal stress, and social values to achieve spatial prioritization in conservation planning (Magris et al., 2017; Whitehead et al., 2014). In this context, highly diverse sites with ecologically unique species (based on traits) are considered of high value (Cadotte & Tucker, 2018). In the case of marine fishes, new global hotspots of biodiversity were highlighted when mapping functional diversity metrics derived from traits (Stuart‐Smith et al., 2013). Specifically for coral reef fish, spatial patterns of functional diversity are increasingly being studied to guide marine protected area planning (Magris et al., 2017). Important conservation dilemmas can arise when spatial patterns of different facets of biodiversity (e.g., taxonomic, functional, and phylogenetic) are incongruent and caution has been raised against using any single facet of biodiversity as a surrogate for others (Cadotte & Tucker, 2018; Devictor et al., 2010b). More integrative approaches are called for, including mapping different facets of biodiversity to reveal complementary information on the location of areas of conservation interest, or the combination of different diversity facets into single prioritization metrics (Cadotte & Tucker, 2018; Devictor et al., 2010b). Here, we compare the ranking of 12 sites according to morphological diversity (i.e., richness and dispersion) when morphology is characterized in terms of traits, landmarks, and outlines. Although, as expected, sites did not rank in the exact identical order when using the different approaches, the overall rankings were reasonably congruent. In consequence, we support the notion of multi‐faceted site‐level prioritization and argue that this may also be useful when considering morphological diversity itself. Mapping morphological diversity levels in terms of traits, landmarks, and outlines, but also using sets of traits related to different ecological functions, may provide complementary information and thus allow for more informed prioritization decisions.

A common objective in ecology is to compare diversity metrics across areas of different environmental conditions. It is therefore important to test whether the outcomes of such comparisons are susceptible to the choice of method used to quantify morphology. We found that most approaches were consistent in concluding that morphological richness did not differ among wave exposure levels. LA was the only approach leading us to conclude that fish morphological dispersion was marginally higher under moderate‐ than under high wave exposure. Morphological dispersion is a function of the distance between the species and the average morphology of the assemblage. Hence, this observation likely responds to the fact that, when using landmarks, species aggregated mostly at the extremes of the morphospace, leaving its center largely unoccupied.

Although morphology of organisms can be measured in more than two dimensions, our study acquired information from photographs, and thus focused on the 2D aspects of shape visible in the lateral view. Using pictures, additional 2D orthogonal views are required to characterize the cross‐section body shape. All three morphometric approaches compared here would benefit from acquiring and analyzing such images (Bouby et al., 2018; Maestri et al., 2018; Price et al., 2019).

TM is reportedly a robust approach to quantify morphology given the well‐documented relationships between morphological traits and functions, such as mobility and feeding (Sibbing & Nagelkerke, 2001; Villéger et al., 2017). Here, we arrive at congruent conclusions using TM, LM, and OA, and argue that the interpretability of the ecological patterns captured by LM and OA would benefit from further research on the links between landmarks—and outlines and functions. Landmarks are easy to identify on homologous points and are therefore amenable to citizen science projects to quantify fish morphology (Chang & Alfaro, 2016). Both TM and LA allow for the selection of traits and landmarks, and thus can be viewed as subjective. Study‐specific selection indeed complicates the comparison of morphological diversity indices across studies using different sets of traits. OA captures aspects of morphology that are undetected by any other approach, such as the caudal fin contour, which distinguished *Zebrasoma* from all other Acanthuridae. OA is remarkably amenable to automation, when pictures are taken on a plain background thus maximizing reproducibility (Hsiang et al., 2018). This attribute could make morphology studies more comparable and ultimately facilitate the compilation of a global fish outline database.

## CONFLICT OF INTEREST

The authors declare no competing interests.

## AUTHOR CONTRIBUTION

**Marita Quitzau:** Conceptualization (equal); Data curation (lead); Formal analysis (equal); Investigation (equal); Methodology (equal); Software (supporting); Visualization (equal); Writing – original draft (equal). **Romain Frelat:** Conceptualization (equal); Data curation (supporting); Formal analysis (equal); Investigation (equal); Methodology (equal); Resources (lead); Software (lead); Supervision (equal); Validation (equal); Visualization (equal); Writing – original draft (equal). **Vincent Bonhomme:** Software (supporting); Validation (equal); Writing – original draft (equal). **Christian Möllmann:** Funding acquisition (equal); Supervision (equal); Writing – original draft (equal). **Leopold Nagelkerke:** Funding acquisition (equal); Supervision (equal); Writing – original draft (equal). **Sonia Bejarano:** Conceptualization (lead); Data curation (supporting); Formal analysis (equal); Investigation (equal); Methodology (equal); Supervision (equal); Validation (equal); Visualization (equal); Writing – original draft (lead).

### OPEN RESEARCH BADGES

This article has earned an Open Data and Open Materials Badge for making publicly available the digitally‐shareable data necessary to reproduce the reported results. The data is available at https://doi.org/10.5281/zenodo.5909813 and https://rfrelat.github.io/CoralFishes.

## Supporting information

Supinfo S1

## Data Availability

We provide the script and dataset on GitHub, together with a tutorial explaining the different steps to apply and compare the three morphometrics approaches (https://rfrelat.github.io/CoralFishes). All data and code necessary to reproduce the results and figures of this study are freely available on Zenodo: https://doi.org/10.5281/zenodo.5909813.
