# Supplementary material for: Traits, landmarks and outlines: Three congruent sides of a tale on coral reef fish morphology
Source: Ecol Evol. 2022 Apr 21;12(4):e8787. doi: 10.1002/ece3.8787 (PMC9021933; doi:10.1002/ece3.8787)
Supplement: Supplementary file 1 — Supinfo S1 [file ECE3-12-e8787-s001.docx]

Supplementary information to

**Traits, landmarks and outlines: Three congruent sides of a tale on coral reef fish morphology**

Marita Quitzau, Romain Frelat, Vincent Bonhomme, Christian Möllmann, Leo Nagelkerke, Sonia Bejarano

*Content*

- **Figure S1**. Morphometric characteristics considered in the traditional morphometrics approach (TM).
- **Table S1**. Calculation of morphological traits from morphometric characteristics.
- **Figure S2**. Landmarks used in geometric morphometrics approach (LA).
- **Figure S3**. Steps of outline analysis approach (OA).
- **Table S2**. Feeding functional groups that differ in *how* fish feed and *what* they feed on, and their ecological significance.
- **Table S3**. Classification of reef sites (Palau) in three levels of wave exposure.
- **Figure S4**. Morphological differentiation of eleven genera within morphospaces.
- **Figure S5**. Morphological differentiation of the genus *Zebrasoma* among the Acanthuridae*.*
- **Table S4**. Morphological diversity of herbivores across wave exposure levels (ANOVA outputs).
- **Table S5**. Subset of more overlapping traits and landmarks used for ancillary analyses.
- **Figure S6**. Axes of morphological variation across individuals when using more overlapping subsets of traits and landmarks.
- **Figure S7**. Pairwise correlation coefficients between individuals‘scores on PC1-PC3 obtained from TM and LA, when using more overlapping subsets of traits and landmarks.
- **Table S6**. Morphological differentiation among taxonomic and feeding groups measured by silhouette values, when using more overlapping subsets of traits and landmarks.
- **Table S7**. Morphological diversity across reef sites when using more overlapping subsets of traits and landmarks.
- **Figure S8**. Morphological diversity across wave exposure levels when using more overlapping subsets of traits and landmarks.
- **References cited**


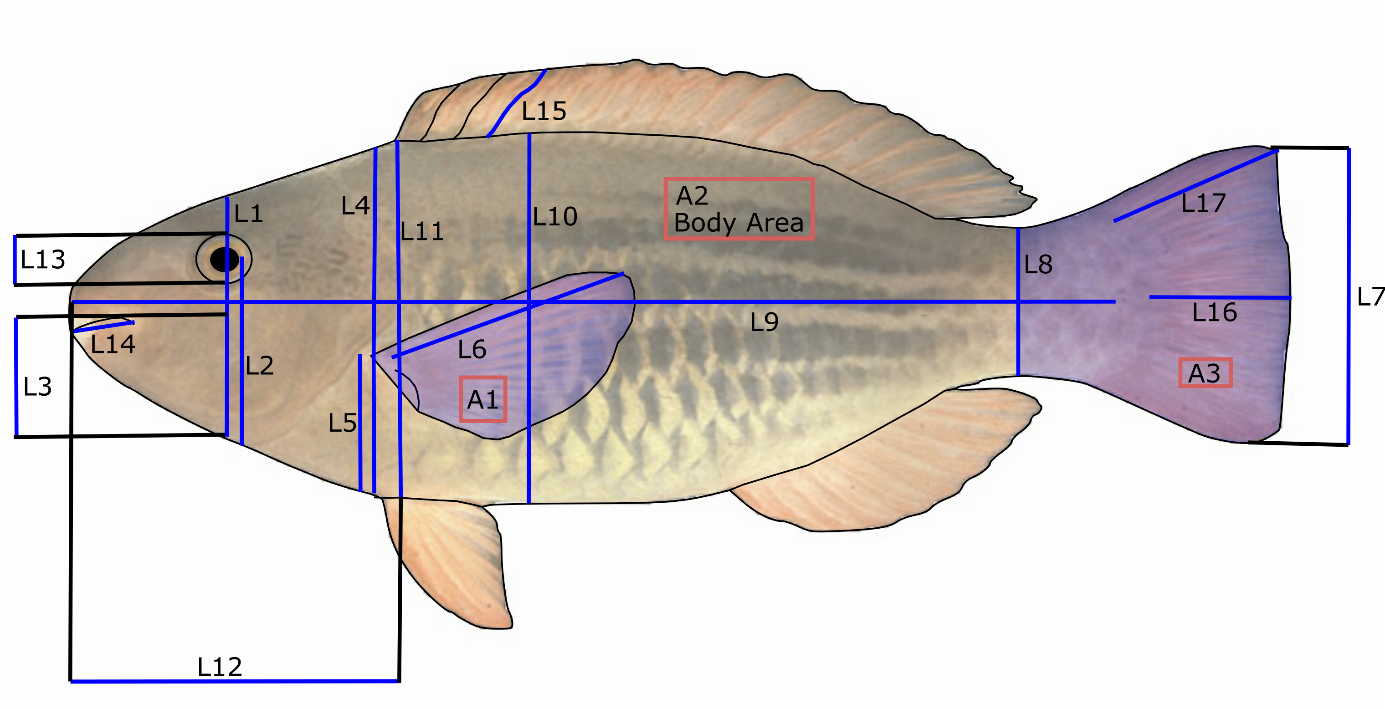
**Figure S1**

| Measurement | Description |
| --- | --- |
| L1 | Head depth along the vertical axis of the eye |
| L2 | Distance from the centre of the eye to the bottom of the head |
| L3 | Distance from the top of the mouth to the bottom of the head along the head depth axis |
| L4 | Body depth at the level of the pectoral fin insertion |
| L5 | Distance between the intersection of the pectoral fin to the bottom of the head |
| L6 | Pectoral fin length |
| L7 | Caudal fin depth |
| L8 | Caudal peduncle minimal depth |
| L9 | Standard length |
| L10 | Maximum Body depth |
| L11 | Head depth |
| L12 | Head length |
| L13 | Eye diameter |
| L14 | Lower jaw length |
| L15 | Longest dorsal spine |
| L16 | Medial caudal fin ray length |
| L17 | Longest caudal fin ray length |
| A1 | Pectoral fin surface |
| A2 | Body area excluding fins to narrowest point on caudal peduncle |
| A3 | Caudal fin surface |

**Figure S1**. Morphometric characteristics used to derive morphological traits in the traditional morphometrics approach (TM). used for the calculation of the morphological traits (Table S1).

**Table S1**

**Table S1.** Morphological traits calculated from the morphometric measurements taken on fish photographs (Figure S1) indicating their reported correlations with fish functions.

| Name (Abbreviation) | Description | Relation to function |
| --- | --- | --- |
| Body aspect ratio (BodyAR) | L10²/A2 | Locomotion, manoeuvrability ^9,10^ |
| Aspect ratio of the caudal fin (CaudalAR) | L7²/A3 | Locomotion, caudal fin is used for propulsion and/or direction ^10,11^ |
| Medial caudal fin ray length (CaudalFL) | L16/L17 | Locomotion ^9,10,12^ |
| Caudal peduncle throttling (CaudalPT) | L7/L8 | Locomotion, caudal propulsion efficiency ^10,11^ |
| Longest dorsal spine (DorsalS) | L15/L10 | Locomotion and defence against predation ^9,13^ |
| Eye position (EyeP) | L2/L1 | Locomotion, vertical position in the water column ^11,14^ |
| Eye size (EyeS) | L13/L1 | Diet, prey detection ^11,15^ |
| Head depth (HeadD) | L11/L10 | Diet and locomotion ^9,16,17^ |
| Head length (HeadL) | L12/L9 | Diet and locomotion ^9,16,17^ |
| Lower jaw length (LJawL) | L14/L12 | Diet ^9,13,18^ |
| Oral gape position (OralGP) | L3/L1 | Diet, feeding method in the water column ^11,19^ |
| Aspect ratio of the pectoral fin (PectoAR) | L6²/A1 | Locomotion, pectoral fin use for propulsion ^11,20^ |
| Pectoral fin position (PectoP) | L5/L4 | Locomotion, pectoral fin use for manoeuvrability ^11,21^ |

**Figure S2**


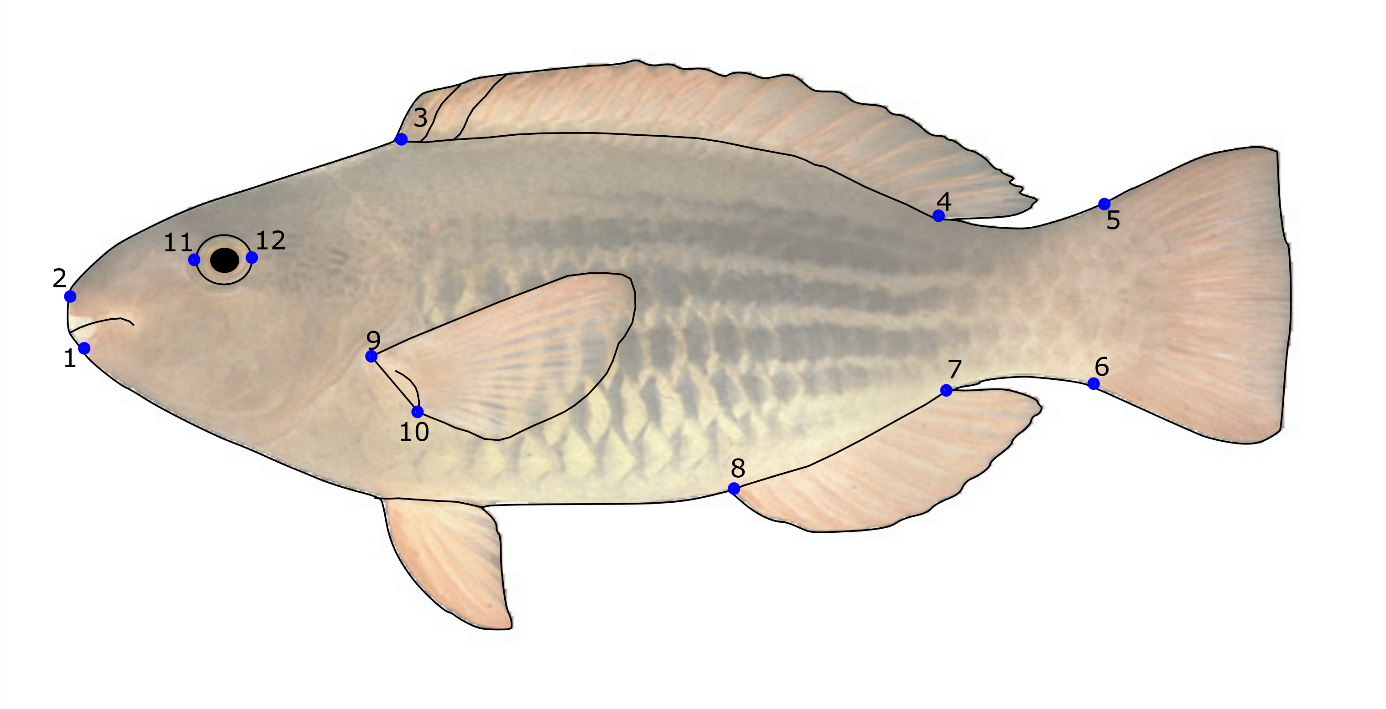


| Landmark | Description |
| --- | --- |
| 1 | Posterior-ventral margin of the distal arm of the maxilla |
| 2 | Most anterior proximal limit between the premaxilla and the head of the maxilla |
| 3 | Insertion of the most anterior ray of the dorsal fin on the body |
| 4 | Insertion of the most posterior ray of the dorsal fin on the body |
| 5 | Dorsal insertion of the caudal fin |
| 6 | Ventral insertion of the caudal fin |
| 7 | Insertion of the first ray of the anal fin |
| 8 | Insertion of the last ray of the anal fin |
| 9 | Anterior-dorsal insertion of the pectoral fin |
| 10 | Posterior-ventral insertion of the pectoral fin |
| 11 | Most anterior point on the eye |
| 12 | Most posterior point on the eye |

**Figure S2.** Landmarks used in the geometric morphometrics approach (LA).

**Figure S3**


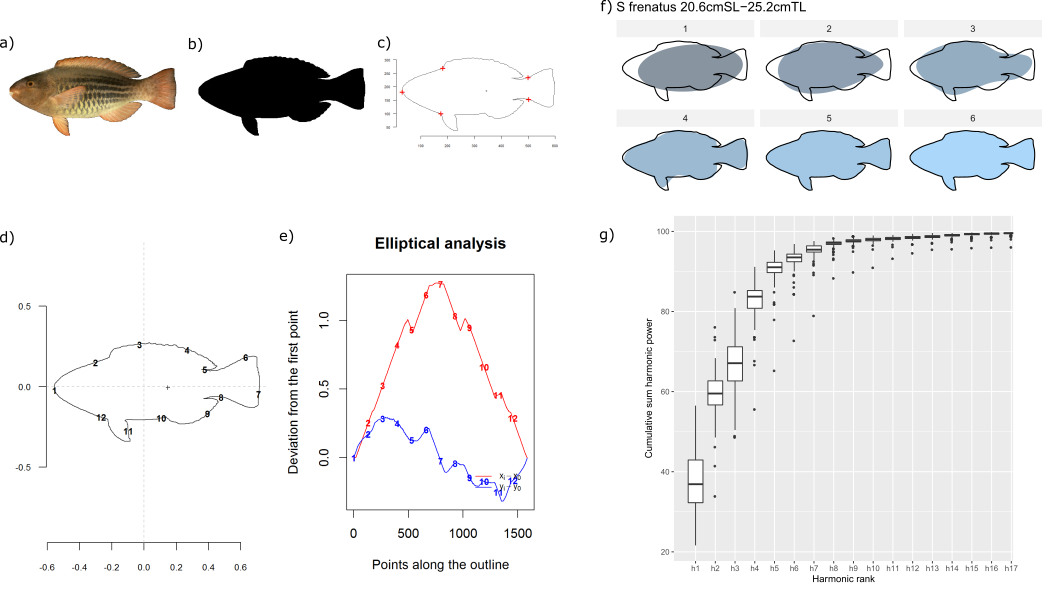


**Figure S3**. Steps of outline analysis approach (OA). The procedure starts with (a) the raw image being transformed into (b) a black and white image in GIMP, which is (c) loaded in R as an outline. (d) The contour of the fish is seen as two periodic functions (x and y coordinates) which are (e) transformed using EFT. (f) Cumulative sum of harmonic power as a function of the harmonic rank indicating the percentage of explained shape per individual by representing the mean and standard deviation, as well as, outliers. (g) Representation of described shape with 1, 2, 4, 15 or 20 harmonics, based on *Scarus frenatus* as an example.

**Table S2**

**Table S2.** Feeding groups of nominally herbivorous fish that differ in foraging behaviour and the type interaction with different components of the reef benthos.

| **Traits** | **Definition, scale or values** | **Relation to ecosystem function** |
| --- | --- | --- |
| Interaction with the calcareous reef matrix | **Excavators**: excavate the substrate with every bite consuming reef carbonates along with epilithic and endolithic organisms.  **Scrapers**: scrape the substrate with every bite mostly feeding on filamentous turf algae and associated detritus.  **Non-bioeroders**: do not interact with the calcareous reef matrix when feeding. | Behavioural trait that reflects the species’ capacity to provide areas of clean calcareous substratum for benthic colonisation, or their contribution to reef bioerosion and production of calcareous sediment ^5^. |
| Interaction with  algal turfs | **Removers**: remove all turfs in one bite. **Croppers**: crop parts of turfs.  **Farmers** (intensive): aggressively defend small territories in which they weed intensively, cultivating monospecific turfs and excluding macroalgae.  **Non-consumers**: do not consume algal turfs. | Trophic trait that reflects the species' capacity reduce algal height, thus decreasing its capacity to retain sediments and improving the suitability of benthic space for larval recruitment ^1^. Informative of the capacity of species to help reefs resist shifts to alternate states ^2^ or to substantially modify the structure of algal communities ^3,4^. |
| Interaction with upright macroalgae | **Macroalgal browsers:** upright macroalgae are their main diet component and browsing their main feeding mode.  **Incidental consumers**: upright macroalgae have been recorded in their diet but are not the dominant or sole component.  **Non-consumers**: have not been recorded feeding on macroalgae, or no upright macroalgae have been detected in their diet. | Trophic trait informative of the species’ capacity to remove macroalgae, and thus potentially contribute to the reversal of coral-to-macroalgal dominance shifts ^6^. |

**Table S3**

**Table S3.** Classification of reef sites on Uchelbeluu reef (Palau) in three levels of wave exposure

| Wave exposure level | Sites |
| --- | --- |
| Low (0.9-26.6 J m^-3^) | C, D, E, F |
| Moderate (46.7 - 68.7 J m^-3^) | A, B, G, H, I |
| High (~221 J m^-3^) | J, K, L |

**Figure S4**


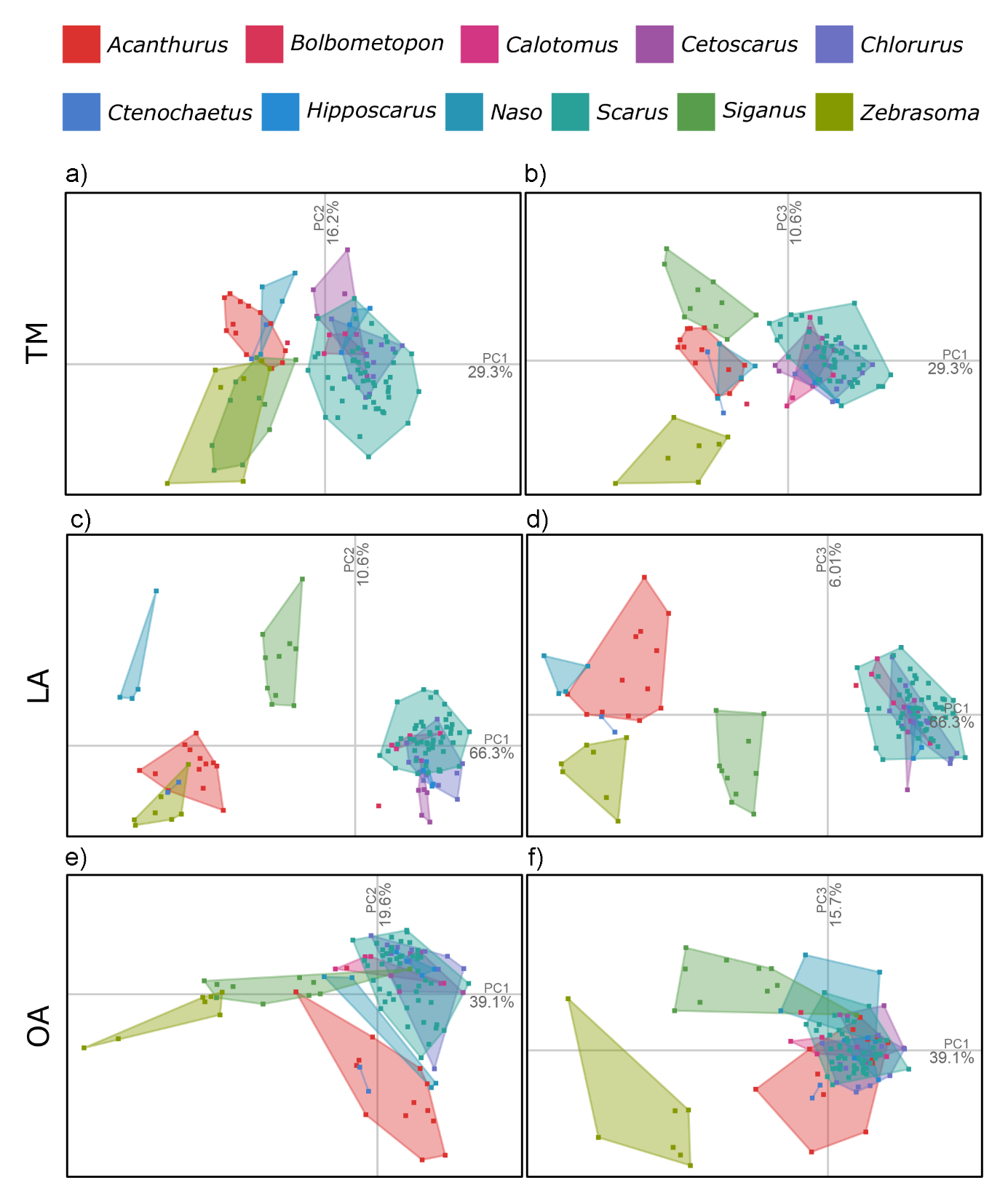


**Figure S4.** Distribution of the eleven genera within morphospaces defined by traits using TM (a-b), landmarks using LA (c-d), and outlines using OA (e-f). Dots are individual fish ordered on PCs 1 and 2 (a, c, e) and PCs 1 and 3 (b, d, f), colour coded and enclosed in separate convex hulls by genus.

**Figure S5**

**
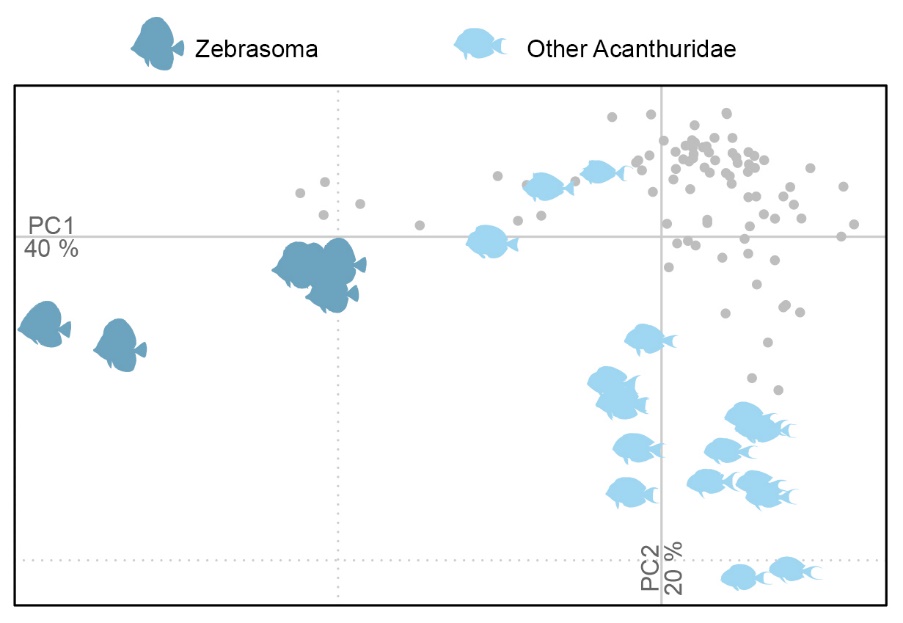
**

**Figure S5.** Separation of individuals of the genus Zebrasoma (dark purple) from other Acanthuridae (light purple) within the morphospace defined by outlines (OA). Grey dots represent individual Siganidae and Labridae (Scarinae).

**Table S4**

**Table S4.** Morphological diversity of herbivores across wave exposure levels. Outputs of ANOVAs comparing morphological richness and dispersion across reef sections subject to low, moderate, and high wave exposure on Uchelbeluu reef (Palau). ANOVAs indicating a significant effect of wave exposure were followed by a Tukey test to test for pairwise differences. Pairwise differences (in this case marginally significant) are highlighted using bold underlined text.

|  | **Outputs of ANOVAs comparing wave exposure levels** | | | |
| --- | --- | --- | --- | --- |
| **Morphological diversity metric** | Estimate | Std. Error | t value | Pr(>\|t\|) |
| Functional Richness (TM) |  |  |  |  |
| Intercept (High wave exposure) | 2.466 | 0.960 | 2.569 | 0.030 |
| Low wave exposure | 1.803 | 1.270 | 1.420 | 0.189 |
| Moderate wave exposure | 0.477 | 1.214 | 0.392 | 0.704 |
| Adjusted R squared |  |  |  | 0.030 |
| p- value |  |  |  | 0.354 |
| Functional Richness (LA) |  |  |  |  |
| Intercept (High wave exposure) | 3.633 | 1.433 | 2.535 | 0.032 |
| Low wave exposure | 0.818 | 1.896 | 0.432 | 0.676 |
| Moderate wave exposure | 1.735 | 1.813 | 0.957 | 0.364 |
| Adjusted R squared |  |  |  | -0.106 |
| p- value |  |  |  | 0.638 |
| Functional Richness (OA) |  |  |  |  |
| Intercept (High wave exposure) | 4.570 | 2.247 | 2.034 | 0.073 |
| Low wave exposure | 1.293 | 2.973 | 0.435 | 0.674 |
| Moderate wave exposure | 1.527 | 2.842 | 0.537 | 0.604 |
| Adjusted R squared |  |  |  | -0.182 |
| p- value |  |  |  | 0.859 |
| Functional Dispersion (TM) |  |  |  |  |
| Intercept (High wave exposure) | 1.200 | 0.070 | 17.088 | 0.000 |
| Low wave exposure | 0.179 | 0.093 | 1.931 | 0.086 |
| Moderate wave exposure | 0.107 | 0.089 | 1.208 | 0.258 |
| Adjusted R squared |  |  |  | 0.136 |
| p- value |  |  |  | 0.210 |
| Functional Dispersion (LA) |  |  |  |  |
| Intercept (High wave exposure) | 1.308 | 0.072 | 18.224 | 0.000 |
| Low wave exposure | 0.209 | 0.095 | 2.196 | 0.056 |
| Moderate wave exposure | 0.265 | 0.091 | 2.924 | 0.017 |
| Adjusted R squared |  |  |  | 0.384 |
| p- value |  |  |  | 0.046 |
| Low vs. high wave exposure | 0.209 | 0.095 | 2.196 | 0.125 |
| Moderate vs. high wave exposure | 0.265 | 0.091 | 2.924 | 0.041 |
| Moderate vs. low wave exposure | 0.057 | 0.083 | 0.683 | 0.779 |
| Functional Dispersion (OA) |  |  |  |  |
| Intercept (High wave exposure) | 1.278 | 0.134 | 9.546 | 0.000 |
| Low wave exposure | 0.272 | 0.177 | 1.534 | 0.160 |
| Moderate wave exposure | 0.322 | 0.169 | 1.901 | 0.090 |
| Adjusted R squared |  |  |  | 0.145 |
| p- value |  |  |  | 0.201 |

**Table S5**

**Table S5.** Subset of more overlapping traits and landmarks used in ancillary analyses (Figs. S6 - S8 and Tables S6-S7).

| **Traits** | **Landmarks** |
| --- | --- |
| 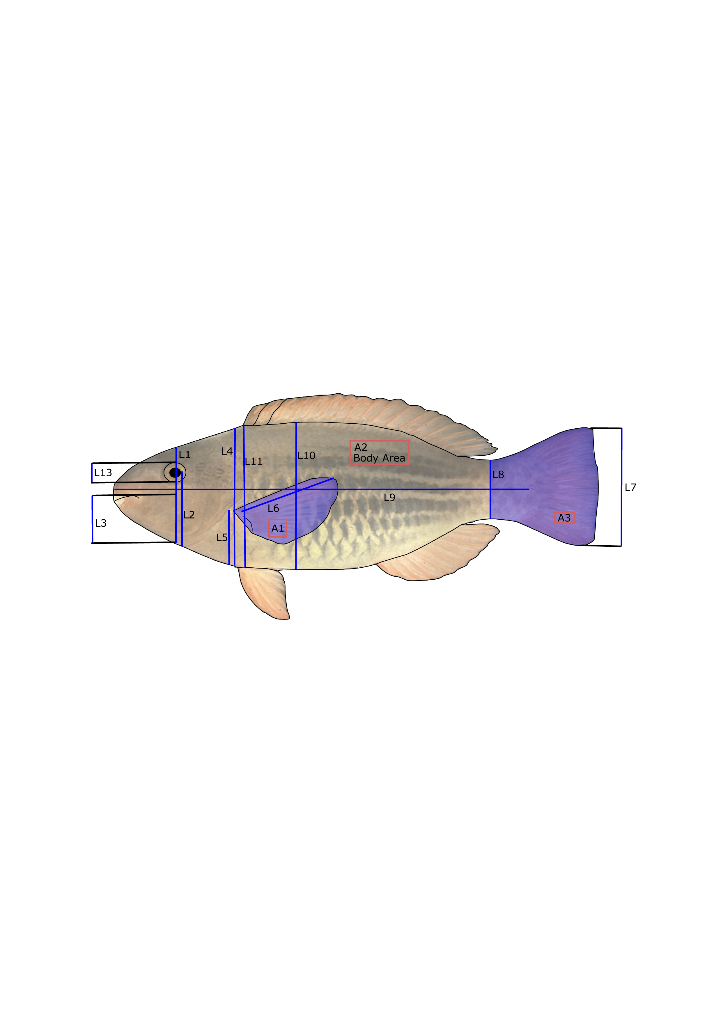 | 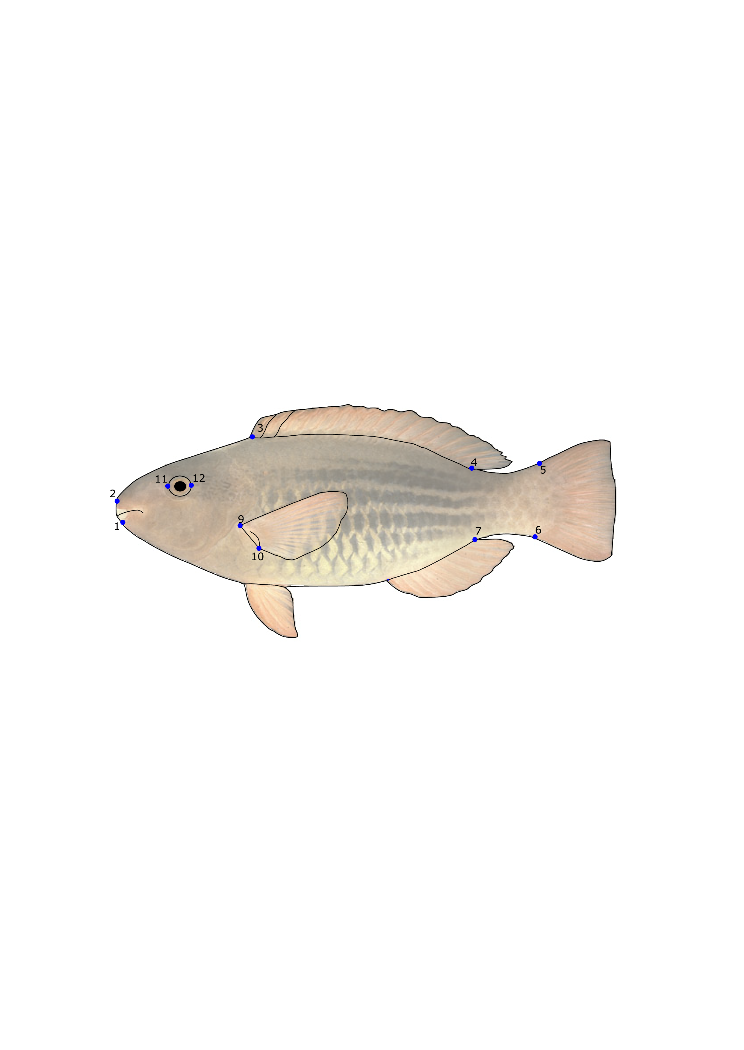 |
| Body aspect ratio (BodyAR) | 1. Posterior-ventral margin of the distal arm of the maxilla |
| Aspect ratio of the caudal fin (CaudalAR) | 2. Most anterior proximal limit between the premaxilla and the head of the maxilla |
| Caudal peduncle throttling (CaudalPT) | 3. Insertion of the most anterior ray of the dorsal fin on the body |
| Eye position (EyeP) | 4. Insertion of the most posterior ray of the dorsal fin on the body |
| Eye size (EyeS) | 5. Dorsal insertion of the caudal fin |
| Head depth (HeadD) | 6. Ventral insertion of the caudal fin |
| Head length (HeadL) | 7. Insertion of the first ray of the anal fin |
| Oral gape position (OralGP) | 9. Anterior-dorsal insertion of the pectoral fin |
| Aspect ratio of the pectoral fin (PectoAR) | 10. Posterior-ventral insertion of the pectoral fin |
| Pectoral fin position (PectoP) | 11. Most anterior point on the eye |
|  | 12. Most posterior point on the eye |

To further validate our comparisons, ancillary analyses were conducted on subsets of more overlapping traits and landmarks (Table S5). Using these subsets caused no major change in our conclusions. In TM, for instance, the traits that strongly influence individual scores on PC1 and PC2 (i.e. BodyAR, CaudalPT, HeadL) were conserved when using either the full set or subset of traits (Fig. S6). Excluding the insertion point of the anal fin in LA, PC1 explained 16% less of the morphological variability across individuals than using the full set of landmarks. However, differences in elongation, head depth, and caudal peduncle shape continued being major drivers of morphological differentiation along PC1_LA_ (Fig. S6). Based on the subsets of traits and landmarks, TM and LA also identified similar main axes of morphological variation. Individuals’ scores on PC1_TM_ were strongly and positively correlated to individuals’ scores on PC1_LA_ (Pearson correlation coefficient r = 0.87, Fig. S7), and both of these were also correlated with individuals’ scores on PC1_OA_ and PC2_OA_ (r > 0.50, Fig. S7). The strong correlation observed between PC3_TM_, PC2_LA_, and PC3_OA_ using the full set of traits and landmarks, however, disappears when using the subsets. These observations did not change our conclusions regarding the morphological differentiation of families and feeding groups (Table S6) or morphological diversity across sites or wave exposure levels in Palau (Table S7).

**Figure S6**

**
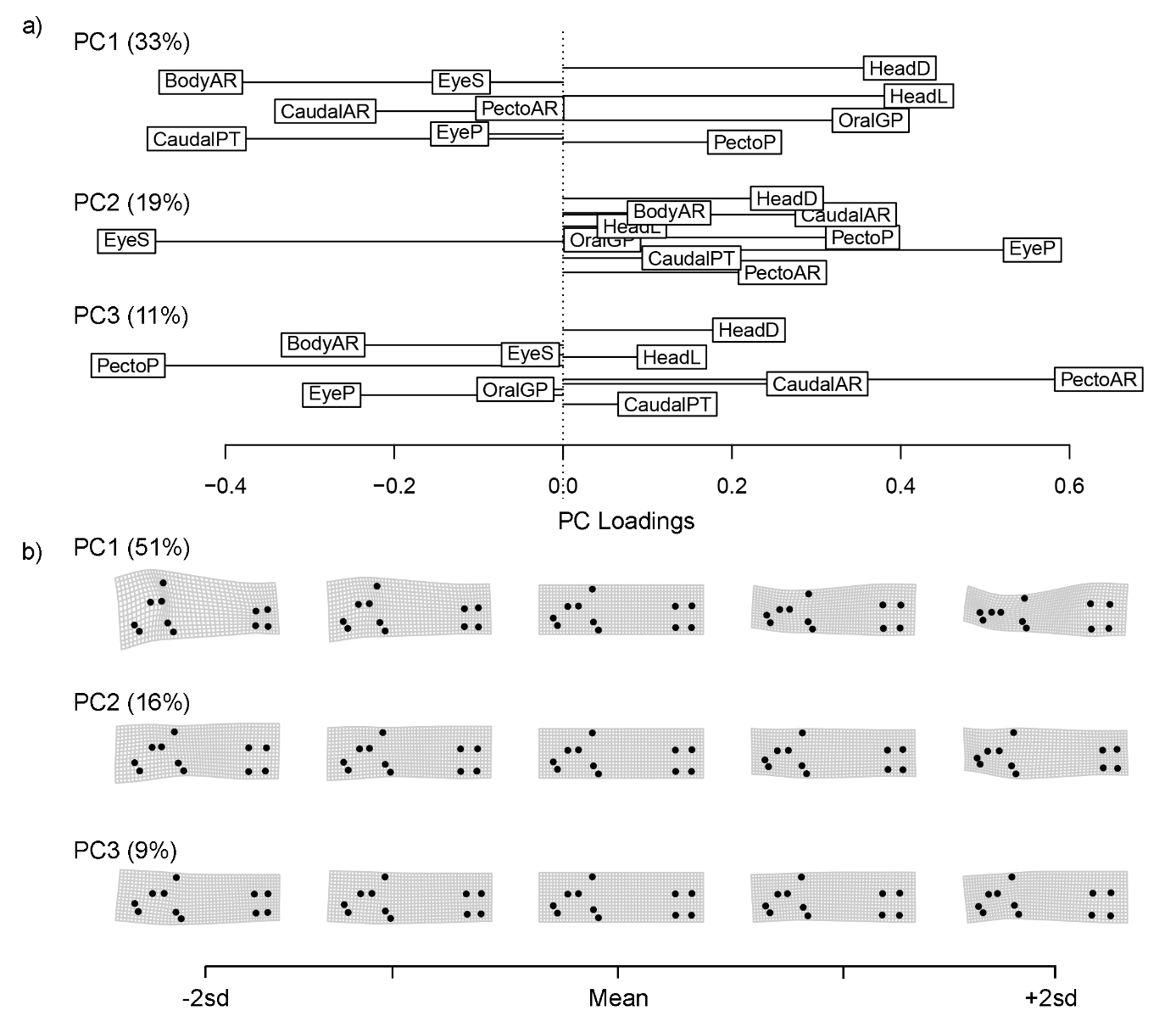
**

**Figure S6.** Diagram indicating the main axes of morphological variation across individual fish when using a subset of more overlapping a) traits and b) landmarks (Table S5). a) Loadings of the morphological traits on the first three PCs of traditional morphometrics (TM). b) Reconstructed position of the landmarks along the first three PCs of landmark analysis (LA).

**Figure S7**

**
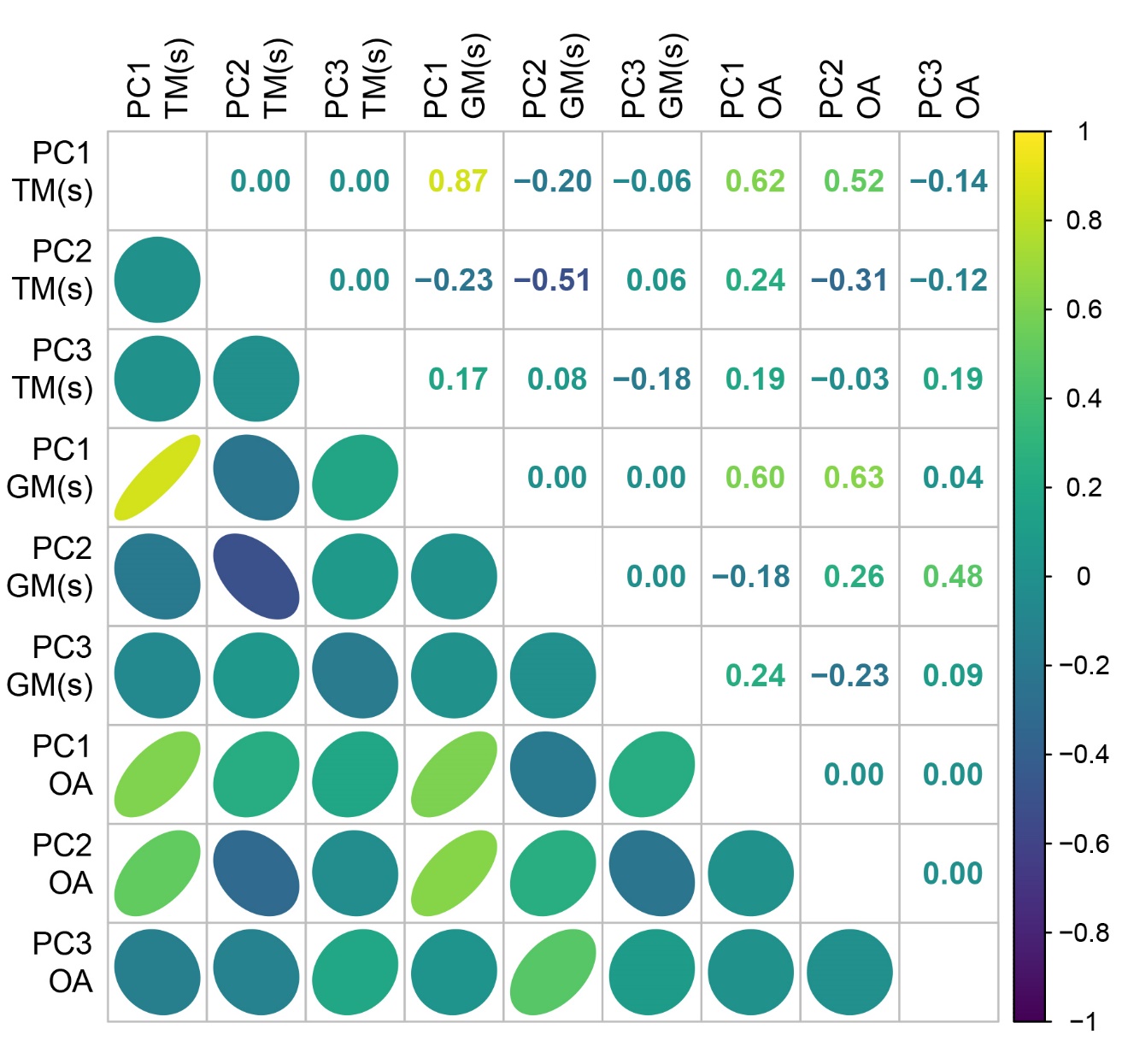
**

**Figure S7.** Grid diagram showing the pairwise Pearson correlation coefficients (top right) between individual fish scores on the first three PCs obtained from TM and LA, when using more overlapping subsets (s) of traits and landmarks (Table S5). Ellipses (bottom left) represent the distribution of the observations. Colours indicate the direction of the correlation (i.e. yellow for positive and blue for negative). Both the shape and colour intensity of the ellipses reflect the value of the Pearson correlation coefficients (i.e. narrowest and darkest ellipses correspond to the highest correlation coefficients, whereas widest and lightest ellipses mark the lowest correlation coefficients).

**Table S6**

**Table S6.** Morphological differentiation among taxonomic and feeding groups measured by silhouette values, when using more overlapping subsets of traits and landmarks (Table S5). Significance is indicated by asterisks and is based on *p-*values (in parentheses) estimated from a null model with 1000 repetitions and adjusted for multiple testing.

| Approach | TM | LA | OA |
| --- | --- | --- | --- |
| **Taxonomic groups** | | | |
| Acanthuridae, Labridae (Scarinae), Siganidae | 0.39 (0.00) * | 0.60 (0.00) * | 0.43 (0.00) * |
| *Acanthurus*, *Bolbometopon*, *Calotomus*, *Cetoscarus*, *Chlorurus*, *Ctenochaetus*, *Hipposcarus*, *Naso*, *Scarus*, *Siganus*, *Zebrasoma* | -0.07 (0.00) * | 0.11 (0.00) * | -0.13 (0.00) * |
|  | | | |
| **Interaction with calcareous reef matrix** (Bioerosion) (excavators, scrapers, non-bioeroders) | 0.12 (0.00) * | 0.29 (0.00) * | 0.01 (0.13) |
| **Interaction with algal turfs** (Algal turf removal)  (intensive farmers, croppers, removers, non-consumers) | 0.19 (0.00) * | 0.32 (0.00) * | 0.10 (0.01) * |
| **Interaction with upright macroalgae**  (macroalgal browsers, incidental consumers, non-consumers) | -0.09 (0.65) | -0.17 (0.73) | -0.19 (0.73) |

**Table S7**

**Table S7.** Palau reef sites A-L, ranked from high to low according to herbivore morphological richness and dispersion when using TM, LA, and OA and more overlapping subsets of traits and landmarks (Table S5).

| **Morphological richness** | | | | | | **Morphological dispersion** | | | | | |
| --- | --- | --- | --- | --- | --- | --- | --- | --- | --- | --- | --- |
| **TM** | | **LA** | | **OA** | | **TM** | | **LA** | | **OA** | |
| B | 7.57 | H | 7.44 | D | 12.89 | B | 1.66 | A | 1.68 | F | 1.82 |
| E | 7.03 | A | 7.26 | A | 9.04 | H | 1.56 | H | 1.65 | D | 1.78 |
| D | 5.92 | B | 6.78 | B | 8.18 | E | 1.51 | B | 1.63 | A | 1.76 |
| L | 5.18 | L | 6.25 | H | 8.10 | I | 1.48 | F | 1.61 | B | 1.72 |
| J | 4.69 | D | 6.24 | L | 7.08 | J | 1.48 | D | 1.60 | H | 1.71 |
| H | 4.11 | E | 6.06 | F | 6.77 | A | 1.47 | L | 1.53 | G | 1.53 |
| A | 4.01 | F | 4.39 | J | 4.42 | L | 1.47 | G | 1.45 | C | 1.43 |
| I | 3.77 | I | 3.65 | I | 2.83 | G | 1.41 | I | 1.42 | L | 1.42 |
| F | 3.52 | J | 3.23 | E | 2.63 | D | 1.39 | E | 1.41 | J | 1.29 |
| K | 2.50 | K | 2.31 | G | 2.34 | F | 1.35 | C | 1.39 | I | 1.28 |
| C | 1.55 | C | 2.09 | K | 2.21 | C | 1.34 | K | 1.31 | E | 1.17 |
| G | 1.53 | G | 1.85 | C | 1.16 | K | 1.26 | J | 1.30 | K | 1.12 |

**Figure S8**

**
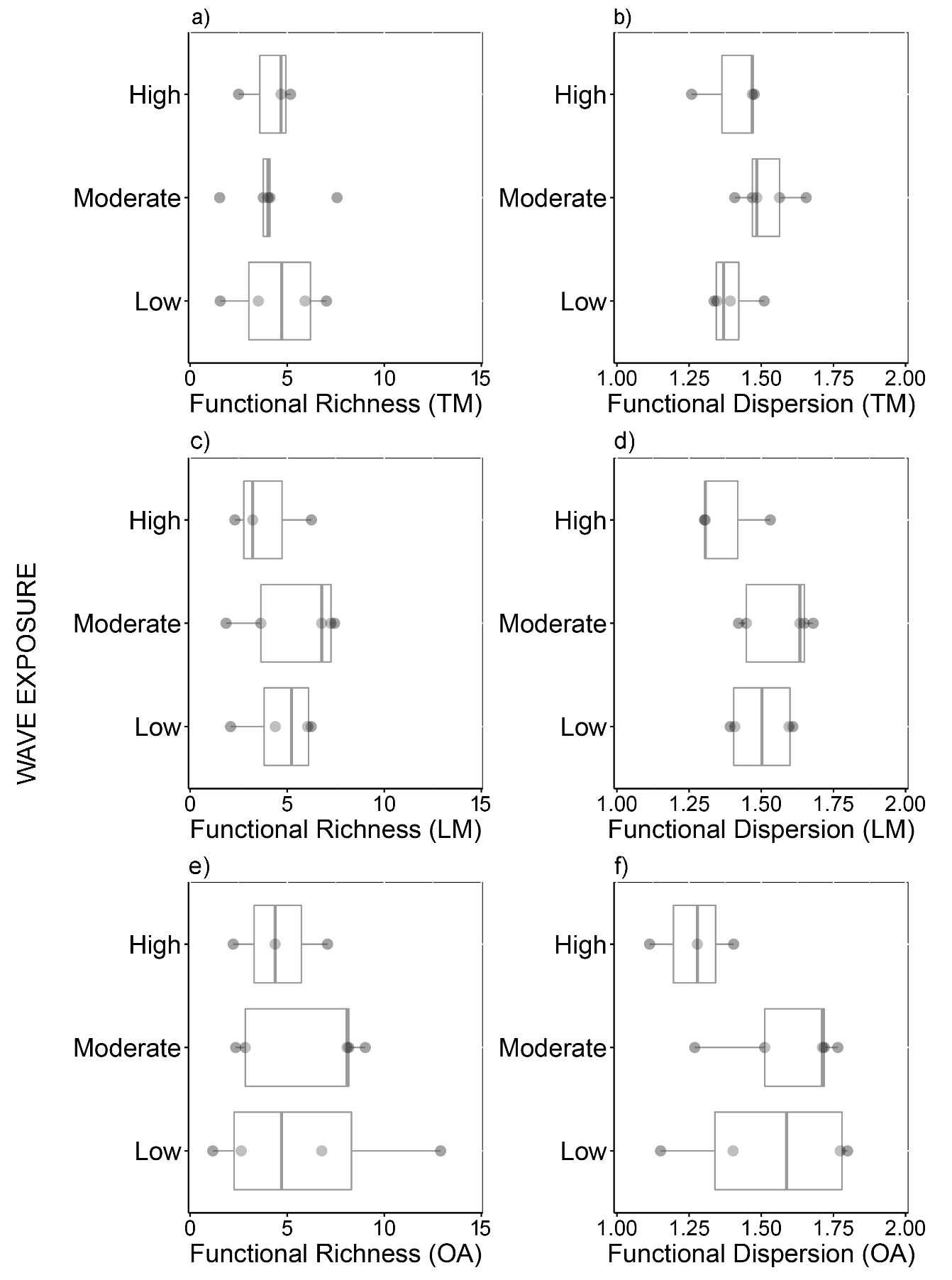
**

**Figure S8.** Herbivore morphological diversity across wave exposure levels when using more overlapping subsets of traits and landmarks (Table S5). Mean (±SE) morphological richness (a, c, e) and morphological dispersion (b, d, f) derived using traits (TM), landmarks (LM), and outlines (OA) in reef areas subject to low, moderate, and high wave exposure on Uchelbeluu reef (Palau).

**References cited**

1. Purcell, S. & Bellwood, D. Spatial patterns of epilithic algal and detrital resources on a windward coral reef. *Coral Reefs* **20**, 117–125 (2001).

2. Hoey, A. S. & Bellwood, D. R. Limited Functional Redundancy in a High Diversity System: Single Species Dominates Key Ecological Process on Coral Reefs. *Ecosystems* **12**, 1316–1328 (2009).

3. Ceccarelli, D. M. Modification of benthic communities by territorial damselfish: A multi-species comparison. *Coral Reefs* **26**, 853–866 (2007).

4. Emslie, M. J. *et al.* Regional-scale variation in the distribution and abundance of farming damselfishes on Australia’s Great Barrier Reef. *Mar. Biol.* **159**, 1293–1304 (2012).

5. Bellwood, D. Direct estimate of bioerosion by two parrotfish species on the Great Barrier Reef, Australia. *Mar. Biol.* **121**, 419–429 (1995).

6. Bellwood, D. R., Hoey, A. S., Ackerman, J. L. & Depczynski, M. Coral bleaching, reef fish community phase shifts and the resilience of coral reefs. *Glob. Chang. Biol.* **12**, 1587–1594 (2006).

7. Diaz-Pulido, G., Harii, S., McCook, L. J. & Hoegh-Guldberg, O. The impact of benthic algae on the settlement of a reef-building coral. *Coral Reefs* **29**, 203–208 (2010).

8. Brandl, S. J. & Bellwood, D. R. Morphology, sociality, and ecology: Can morphology predict pairing behavior in coral reef fishes? *Coral Reefs* **32**, 835–846 (2013).

9. Bellwood, D. R., Goatley, C. H. R., Brandl, S. J. & Bellwood, O. Fifty million years of herbivory on coral reefs: fossils, fish and functional innovations. *Proc. R. Soc. B Biol. Sci.* **281**, 20133046–20133046 (2014).

10. Webb, P. W. Body Form, Locomotion and Foraging in Aquatie Vertebrates. *Am. Zool.* **24**, 107–120 (1984).

11. Villéger, S., Miranda, J. R., Hernandez, D. F. & Mouillot, D. Contrasting changes in taxonomic vs . functional diversity of tropical fish communities after habitat degradation. *Ecol. Appl.* **20**, 1512–1522 (2010).

12. Fisher, R. & Hogan, J. D. Morphological predictors of swimming speed: a case study of pre-settlement juvenile coral reef fishes. *J. Exp. Biol.* **210**, 2436–2443 (2007).

13. Wainwright, P. C., Bellwood, D. R. & Westneat, M. W. Ecomorphology of locomotion in labrid fishes. *Environ. Biol. Fishes* **65**, 47–62 (2002).

14. Gatz, A. J. Community Organization in Fishes as Indicated by Morphological Features. *Ecology* **60**, 711–718 (1979).

15. Boyle, K. S. & Horn, M. H. Comparison of feeding guild structure and ecomorphology of intertidal fish assemblages from central California and central Chile. *Mar. Ecol. Prog. Ser.* **319**, 65–84 (2006).

16. Collar, D. C., Wainwright, P. C. & Alfaro, M. E. Integrated diversification of locomotion and feeding in labrid fishes. *Biol. Lett.* **4**, 84–86 (2008).

17. Li, D., Hu, W., Wang, Y., Zhu, Z. & Fu, C. Reduced swimming abilities in fast-growing transgenic common carp Cyprinus carpio associated with their morphological variations. *J. Fish Biol.* **74**, 186–197 (2009).

18. Goatley, C. & Bellwood, D. Biologically mediated sediment fluxes on coral reefs: sediment removal and off-reef transportation by the surgeonfish Ctenochaetus striatus. *Mar. Ecol. Prog. Ser.* **415**, 237–245 (2010).

19. Sibbing, F. A. & Nagelkerke, L. A. J. Resource partitioning by Lake Tana barbs predicted from fish morphometrics and prey characteristics. 393–437 (2001).

20. Fulton, C. J., Bellwood, D. R. & Wainwright, P. C. The relationship between swimming ability and habitat use in wrasses (Labridae). *Mar. Biol.* **139**, 25–33 (2001).

21. Dumay, O., Tari, P. S., Tomasini, J. a. & Mouillot, D. Functional groups of lagoon fish species in Languedoc Roussillon, southern France. *J. Fish Biol.* **64**, 970–983 (2004).
